# Heme-deficient primitive red blood cells induce HSPC ferroptosis by altering iron homeostasis during zebrafish embryogenesis

**DOI:** 10.1242/dev.201690

**Published:** 2023-06-16

**Authors:** Peng Lv, Feng Liu

**Affiliations:** ^1^ State Key Laboratory of Membrane Biology, Institute of Zoology, Institute for Stem Cell and Regeneration, Chinese Academy of Sciences, Beijing 100101, China; ^2^ University of Chinese Academy of Science, Beijing 100049, China; ^3^ School of Life Sciences, Shandong University, Qingdao 266237, China

**Keywords:** Hematopoietic stem and progenitor cell, Primitive RBC, Iron homeostasis, Ferroptosis, Zebrafish

## Abstract

The crosstalk between hematopoietic lineages is important for developmental hematopoiesis. However, the role of primitive red blood cells (RBCs) in the formation of definitive hematopoietic stem and progenitor cells (HSPCs) is largely unknown. Primitive RBC deficiencies in mammals always lead to early embryonic lethality, but zebrafish lines with RBC deficiencies can survive to larval stage. By taking advantage of a zebrafish model, we find that the survival of nascent HSPCs is impaired in *alas2*- or *alad*-deficient embryos with aberrant heme biosynthesis in RBCs. Heme-deficient primitive RBCs induce ferroptosis of HSPCs through the disruption of iron homeostasis. Mechanistically, heme-deficient primitive RBCs cause blood iron-overload via Slc40a1, and an HSPC iron sensor, Tfr1b, mediates excessive iron absorption. Thus, iron-induced oxidative stress stimulates the lipid peroxidation, which directly leads to HSPC ferroptosis. Anti-ferroptotic treatments efficiently reverse HSPC defects in *alas2* or *alad* mutants. HSPC transplantation assay reveals that the attenuated erythroid reconstitution efficiency may result from the ferroptosis of erythrocyte-biased HSPCs. Together, these results illustrate that heme-deficient primitive RBCs are detrimental to HSPC production and may provide potential implications for iron dysregulation-induced hematological malignancies.

## INTRODUCTION

The emergence of various hematopoietic lineages occurs through multiple waves of developmental hematopoiesis. In zebrafish, the earliest wave, termed primitive hematopoiesis, mainly generates primitive red blood cells (RBCs) in the intermediate cell mass (ICM) region and some myeloid cells in the cephalic region. Subsequently, the definitive hematopoiesis produces lineage-restricted progenitors and multipotent hematopoietic stem and progenitor cells (HSPCs) through endothelial-to-hematopoietic transition (EHT) in the ventral wall of the dorsal aorta (VDA) (de Jong and Zon, 2005; Kissa and Herbomel, 2010; Orkin and Zon, 2008). A better understanding of HSPC production *in vivo* under physiological and pathological conditions is crucial for HSPC induction *in vitro* and therapeutic applications. Primitive hematopoietic cells are known to provide a niche during HSPC generation in the aorta-gonad-mesonephros (AGM) region (Gao et al., 2018; Vink et al., 2022). Among them, primitive myeloid cells involved in innate immune response have been demonstrated to be important in HSPC production (Espín-Palazón et al., 2014; Frame et al., 2020; He et al., 2015; Travnickova et al., 2015). Nevertheless, the role of primitive RBCs in the production of definitive HSPCs under normal or stress conditions remains elusive.

During embryogenesis, primitive RBCs circulate transiently as nucleated cells and mediate oxygen and carbon dioxide transportation (Palis, 2014; Qian et al., 2007). At the same time, iron homeostasis is also tightly regulated by RBCs during the erythropoiesis (Davuluri et al., 2016; Kautz and Nemeth, 2014). Several RBC-specific genes have been shown to play an important role in primitive erythropoiesis as well as in iron homeostasis. For example, loss of *transcriptional intermediary factor 1 gamma* (*tif1γ*; also known as *trim33*) results in primitive RBC apoptosis and severe aplasia (Ransom et al., 2004); *gata1* (*gata1a*)-deficient embryos show bloodless phenotype without circulating RBCs (Lyons et al., 2002); *kruppel-like factors 1* or *3* (*klf1* or *klf3*) knockdown show low maturity of RBCs (Xue et al., 2015); *alas2* and *alad* mutants were characterized as disease models of congenital sideroblastic anemia (CSA) (Brownlie et al., 1998) and porphyria (Maruno et al., 2001), the corresponding genes encode the first (5'-Aminolevulinate Synthase 2, ALAS2) and second (Aminolevulinate Dehydratase, ALAD) rate-limiting enzymes in the heme biosynthesis pathway (Balwani and Desnick, 2012; Maruno et al., 2001). Recent studies on RBC-related iron disorders (e.g. iron-deficiency or iron-overload) have provided insights into the mechanisms of iron trafficking (Donovan et al., 2005; Jabara et al., 2016), distribution (Nai et al., 2014) and homeostasis regulation (Duarte et al., 2021). Iron plays a dual role during developmental hematopoiesis, in which a moderate amount of iron can facilitate HSPC differentiation and multilineage reconstitution (Bonadonna et al., 2022; Garcia-Prat et al., 2021; Zhang et al., 2022), whereas excessive iron can directly induce cell ferroptosis (Hu et al., 2021; Yu et al., 2020). Furthermore, iron homeostasis is also required for adult HSPC maintenance (Muto et al., 2017; Wang et al., 2020). However, whether and how iron homeostasis affects HSPC formation during embryogenesis is unclear.

In mammals, it is challenging to study whether functional definitive HSPC can be generated in the deficiency of primitive blood cells, owing to the early embryonic lethality (Baron et al., 2012). In contrast, the zebrafish is able to survive to initiate definitive hematopoiesis under the RBC-deficient conditions (Lyons et al., 2002; Ransom et al., 2004). In this study, we examined HSPC development in several zebrafish RBC-deficient models and showed that the HSPC production was impaired only in zebrafish *alas2*- or *alad*-deficient embryos. Our results demonstrate that heme-deficient primitive RBCs induced HSPC ferroptosis through the disruption of iron trafficking, and the iron-ROS-lipid peroxidation axis is responsible for defective HSPC production.

## RESULTS

### HSPC development is impaired in zebrafish *alas2*- or *alad*-deficient embryos

**Fig. 1.**
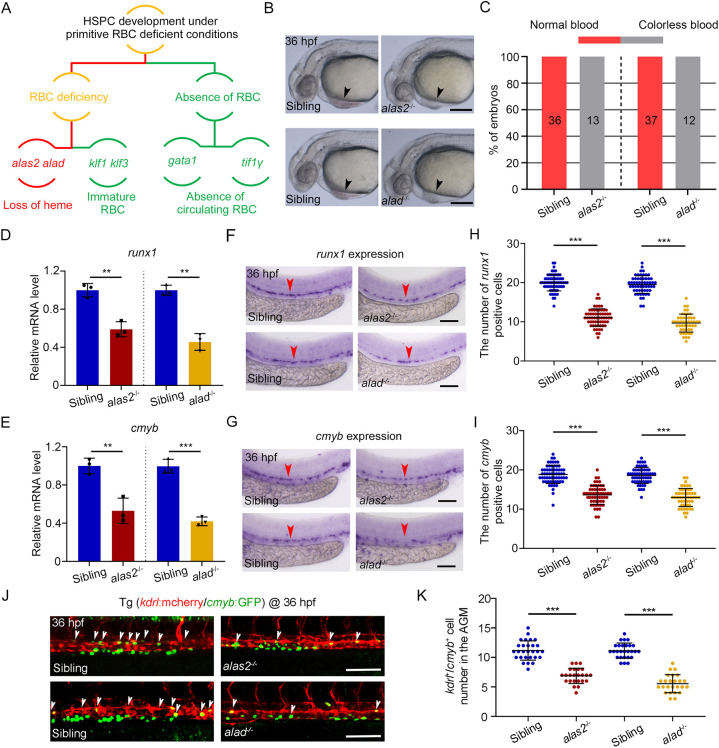
**HSPC formation is impaired in *alas2*- and *alad*-deficient embryos.** (A) Screening strategy of HSPC development under different primitive RBC-deficient conditions. (B) Bright-field images of blood flow in *alas2*^−/−^, *alad*^−/−^ and their siblings at 36 hpf. Blood flow in heart regions is denoted by arrowheads. (C) Cumulative results of blood color phenotype in B. Number of embryos with different blood color are noted in each column. (D,E) Relative mRNA level of *runx1* (D) and *cmyb* (E) in the dissected trunk regions of *alas2*^−/−^, *alad*^−/−^ and their sibling at 36 hpf examined by qRT-PCR. *n*=3 experimental replicates. (F,G) Expression of *runx1* and *cmyb* in *alas2*^−/−^, *alad*^−/−^ and their sibling at 36 hpf examined by WISH. The AGM regions for marker gene-positive cell counting are denoted by red arrowheads. (H,I) Quantification of the *runx1*- and *cmyb*-positive HSPCs in F and G. *n*=3 experimental replicates. (J) Confocal imaging shows the *kdrl*^+^/*cmyb*^+^ HSPCs in AGM regions of *alas2*^−/−^, *alad*^−/−^ and their siblings at 36 hpf. The *kdrl*^+^/*cmyb*^+^ HSPCs in the AGM region are denoted by white arrowheads. (K) Quantification of the indicated cells in confocal imaging. *n*=3 experimental replicates. Number of samples are indicated. Data are mean±s.d. **P<0.01; ***P<0.001 [Mann–Whitney non-parametric *U*-test (D,E); one-way ANOVA, Tukey's multiple comparisons (H,I,K)]. n.s., not significant. Scale bars: 200 μm (B); 100 μm (F,G,J).

In zebrafish, it is widely accepted that the primitive erythropoiesis mainly accounts for the circulating RBCs in the embryos before 4 days post-fertilization (dpf) (Kulkeaw and Sugiyama, 2012), during which the earliest HSPCs emerge in the AGM region. To determine whether the HSPC production is impaired under various primitive RBC-deficient conditions at 36 h post-fertilization (hpf), we phenotypically screened several RBC-deficient models, such as the absence of primitive RBCs (deficiency in *tif1γ* and *gata1*), low-maturity of primitive RBCs (deficiency in *klf1* and *klf3*) and heme-deficient primitive RBCs (deficiency in *alas2* or *alad*) (Fig. 1A). By using morpholino (MO) knockdown of these genes, we found that the number of *runx1*-positive HSPCs in the AGM region was remarkedly decreased only in *alas2*- or *alad*-morphants at 36 hpf (Fig. S1A,B). Confocal microscopy also showed that the number of *kdrl*^+^/*cmyb*^+^ (*myb*^+^) HSPCs was significantly decreased in the AGM region of *alas2*- or *alad*-morphants (Fig. S1C,D).

To validate the observed HSPC deficiency by gene knockdown, we generated *alas2* and *alad* mutants (here refer to *alas2*^−/−^ and *alad*^−/−^) using the CRISPR/Cas9 method. Frameshift mutations were introduced in both mutants (*alas2* mutant, a 1 bp insertion, and *alad* mutant, a 3 bp to 2 bp transition) (Fig. S1E). Both mutations caused the generation of premature stop codons, resulting in truncated proteins (Fig. S1F). In *alas2*^−/−^ or *alad*^−/−^, it showed an evident reduction of *alas2* or *alad* mRNA in the ICM region of primitive RBCs at 24 hpf, respectively, likely due to non-sense mRNA decay (Fig. S1G,H). By using quantitative real-time PCR (qRT-PCR) and western blotting, we detected that both mRNA and protein levels of *alas2* or *alad* were remarkably decreased in *alas2*^−/−^ or *alad*^−/−^ at 36 hpf, respectively (Fig. S1I-L), indicating that the loss-of-function mutations were generated.

As *alas2*^−/−^ and *alad*^−/−^ exhibited a colorless blood phenotype as early as 30 hpf, we were able to morphologically distinguish the homozygous mutant embryos (Fig. 1B,C). We first detected the expression of HSPC marker genes, *runx1* and *cmyb*, by qRT-PCR and whole-mount *in situ* hybridization (WISH) at 36 hpf, and found that both *runx1* and *cmyb* were evidently decreased in *alas2*^−/−^ or *alad*^−/−^ (Fig. 1D-I). Confocal imaging also confirmed that the number of *kdrl*^+^/*cmyb*^+^ HSPCs was also significantly decreased in the AGM region of *alas2*^−/−^ or *alad*^−/−^ at 36 hpf (Fig. 1J,K). To characterize the exact stage of HSPC impairment, we performed time course observation at 28 hpf, 32 hpf and 36 hpf by confocal imaging. The results showed that the specification of homogenic endothelial cells (HECs) was normal at 28 hpf; however, the number of *kdrl*^+^/*cmyb*^+^ HSPCs was decreased after 32 hpf (Fig. S1M,N). Taken together, these results suggest that the HSPC production was impaired in *alas2*- or *alad*-deficient zebrafish embryos after HEC specification.

### Iron accumulates in heme-deficient primitive RBCs

The coding genes of these two heme biosynthesis enzymes, *alas2* and *alad*, are specifically expressed in primitive RBCs at 18, 24 (ICM region) and 36 hpf (circulating RBCs) (Fig. S2A). The loss of Alas2 and Alad results in hemoglobin deficiency due to the heme metabolic anomalies at 36 hpf (Fig. S2B,C). Surprisingly, the number of circulating RBCs in *alas2*^−/−^ or *alad*^−/−^ was normal (Movie 1), and the maturity of primitive RBCs showed no difference in *alas2*^−/−^ or *alad*^−/−^ compared with their siblings at 36 hpf (Fig. S2D,E).

**Fig. 2.**
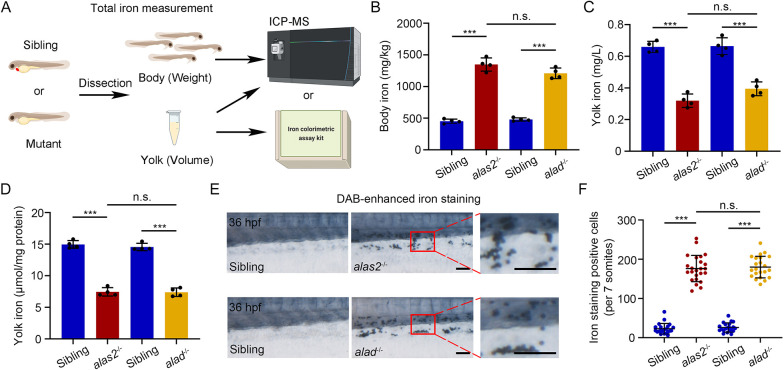
**Heme-deficient primitive RBCs display iron accumulation.** (A) Workflow of iron distribution assay of body and corresponding yolk regions by ICP-MS or iron colorimetric assay kit, respectively, in *alas2*^−/−^, *alad*^−/−^ and their siblings at 36 hpf. (B-D) Quantification of the total iron level of dissected body (B) and yolk (C,D). The body and yolk sac iron content are quantified as weight (mg/kg) and volume (mg/l or μmol/mg protein), respectively. *n*=4 experimental replicates. (E) Representative bright-field images of DAB-enhanced iron staining in *alas2*^−/−^, *alad*^−/−^ and their siblings at 36 hpf. Right column shows magnification of areas in red box showing iron enriched RBCs in caudal hematopoietic tissue (CHT) circulation. (F) Quantification of the iron staining-positive RBCs in E. *n*=3 experimental replicates. Number of samples are indicated. Data are mean±s.d. ***P<0.001 (one-way ANOVA, Tukey's multiple comparisons). n.s., not significant. Scale bars: 100 μm.

Previous studies have showed that Alas2 and Alad levels are closely associated with cellular iron states, and the loss of *alas2* or *alad* in RBCs results in iron accumulation (Abraham et al., 1985; Harigae et al., 2003). To test whether iron metabolism was impaired in the *alas2*^−/−^ or *alad*^−/−^, we detected the expression of *transferrin-a* (*tfa*), which has been proposed to function to deliver yolk iron into blood circulation (Donovan et al., 2000). As expected, both WISH and qRT-PCR results suggested that the mRNA level of *tfa* was significantly increased in the yolk region of *alas2*^−/−^ or *alad*^−/−^ at 36 hpf (Fig. S2F-H), implying that iron metabolism was affected. To determine the dysregulation of iron in *alas2*^−/−^ or *alad*^−/−^, we measured the iron content in dissected zebrafish bodies and yolk using inductively coupled plasma mass spectrometry (ICP-MS) and an iron colorimetric assay kit (Fig. 2A). Interestingly, the results showed that iron content was significantly decreased in the yolk region of both mutants, but increased in the body region (Fig. 2B-D). Next, we examined the body iron distribution by DAB-enhanced iron staining with whole zebrafish embryos at 36 hpf. Bright-field microscopy showed that a large number of iron-enriched RBCs were detected in the circulating blood of *alas2*^−/−^ or *alad*^−/−^ (Fig. 2E,F). Together, these results suggested that iron homeostasis was disrupted in *alas2*^−/−^ or *alad*^−/−^.

### Heme-deficient primitive RBCs induce blood IOL via Slc40a1

**Fig. 3.**
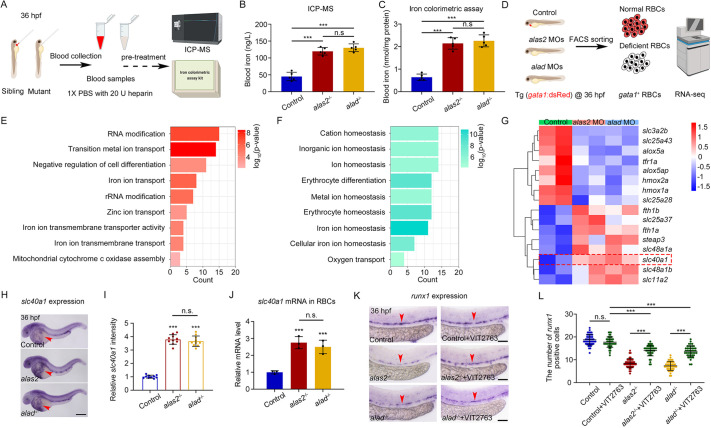
**Blood IOL phenotype is caused by heme-deficient RBCs via Slc40a1.** (A) Workflow of blood iron measurement in control, *alas2*^−/−^ and *alad*^−/−^ at 36 hpf by ICP-MS or iron colorimetric assay kit. (B,C) Quantification of the blood iron content by ICP-MS (B) and iron colorimetric assay kit (C). *n*=5 experimental replicates. (D) Schematic of bulk RNA-seq of flow cytometry-sorted *gata1*^+^ RBCs in *alas2* and *alad* morphants at 36 hpf on Illumina NovaSeq 6000 platform. (E,F) GO analysis showing the enrichment of upregulated (E) and downregulated (F) overlapping terms in heme-deficient RBCs of *alas2* and *alad* morphants. (G) Heatmap analysis of selected overlapping DEGs in iron transportation-related GO terms. The iron exporter Slc40a1 is highlighted with red dashed rectangle. (H) The expression of *slc40a1* is detected by WISH in control, *alas2*^−/−^ and *alad*^−/−^ at 36 hpf. The RBC-enriched yolk regions are denoted by red arrowheads. (I) Statistical analysis of the relative *slc40a1* expression in H by ImageJ. *n* (embryos)=10. (J) Relative mRNA level of *slc40a1* in the flow cytometric-sorted RBCs (*gata1*^+^) of control, *alas2*^−/−^ and *alad*^−/−^ at 36 hpf examined by qRT-PCR. *n*=3 experimental replicates. (K) Expression of HSPC marker gene *runx1* in control, *alas2*^−/−^ and *alad*^−/−^ with or without VIT2763 treatment at 36 hpf examined by WISH. The AGM regions for marker gene-positive cell counting are denoted by red arrowheads. (L) Quantification of the *runx1*-positive HSPCs in K. *n*=3 experimental replicates. Number of samples are indicated. Data are mean±s.d. ***P<0.001 (one-way ANOVA, Tukey's multiple comparisons). n.s., not significant. Scale bars: 250 μm (H); 100 μm (K).

Since the *alas2*- or *alad*-null mouse developed an iron overload (IOL) phenotype (Fleming et al., 2011), and zebrafish *alas2*^−/−^ and *alad*^−/−^ displayed dysregulation of iron in RBCs, we thus sought to determine whether the blood IOL was induced in *alas2*^−/−^ or *alad*^−/−^. To evaluate the blood iron level, we collected blood samples from the heart region in control (mixed *alas2* and *alad* siblings in 1:1 ratio), *alas2*^−/−^ and *alad*^−/−^ at 36 hpf, and the blood iron was measured by ICP-MS and iron colorimetric assay kit (Fig. 3A). We first generated the standard curve of iron assay kit (*y*=0.0791*x+0.0362, R^2^=0.9966). After the measurement, we found that the blood iron was significantly elevated in *alas2*^−/−^ or *alad*^−/−^ at 36 hpf (Fig. 3B,C). At the same time, we confirmed that *alas2*- or *alad*-morphants exhibited the similar blood IOL phenotypes at 36 hpf (Fig. S3A).

To reveal the underlying mechanism by which heme-deficient primitive RBC causes blood IOL, we performed bulk RNA-sequencing (RNA-seq) with flow-cytometry to sort *gata1*^+^ RBCs from control, *alas2*- and *alad*-morphants at 36 hpf (Fig. 3D). A total of 945 overlapping differentially expressed genes (DEGs) were detected in *alas2*- or *alad*-morphant RBCs (Fig. S3B; Table S1) – among them, 613 upregulated and 332 downregulated overlapping DEGs were further characterized, respectively (Fig. S3C). Gene ontology (GO) analyses demonstrated that a group of upregulated genes were enriched in the term of ‘iron transport’, and downregulated genes were found to be primarily enriched in ‘ion homeostasis’ or ‘erythrocyte differentiation’ (Fig. 3E,F). A heatmap of overlapping DEGs showed that the expression of the iron exporter *slc40a1* (also known as *ferroportin*) was upregulated in both *alas2*- or *alad*-morphant RBCs (Fig. 3G). To confirm the upregulation of *slc40a1* mRNA level in heme-deficient RBCs, we performed WISH and qRT-PCR by using *alas2*^−/−^ or *alad*^−/−^ at 36 hpf. As expected, both results suggested that *slc40a1* was significantly upregulated in the RBCs of *alas2*^−/−^ or *alad*^−/−^ at 36 hpf (Fig. 3H-J).

Previous studies have shown that the iron transporter Slc40a1 is required for cellular iron release (Donovan et al., 2005; Fraenkel et al., 2005). To test whether the blood IOL induced by heme-deficient RBCs was Slc40a1 dependent, we utilized a widely used Slc40a1 inhibitor, VIT2763, which blocks iron efflux by inhibiting hepcidin binding to Slc40a1 (Manolova et al., 2019; Richard et al., 2020). After treatment with VIT2763 from 26 to 36 hpf, we detected that in *alas2*^−/−^ or *alad*^−/−^, the iron content in blood and RBCs was significantly reduced and increased, respectively, whereas it was not changed in the yolk region (Fig. S3D-F). These results further confirmed that the blood iron overload was induced by heme-deficient RBCs. Moreover, we detected that the HSPC defects were also restored in *alas2*^−/−^ or *alad*^−/−^ after VIT2763 treatment (Fig. 3K,L). Due to the limited number of mature hepatocytes and macrophages at 36 hpf of zebrafish embryos, we thus speculated that the numerous heme-deficient primitive RBCs but not hepatocytes or macrophages contributed to the blood IOL via Slc40a1.

To further test whether the HSPC defects were heme-deficient RBC-dependent in *alas2*^−/−^ and *alad*^−/−^, we used *gata1* MO to block the production of abnormal RBCs (Fig. S3G). We first detected that blood IOL was significantly alleviated in *alas2*^−/−^ or *alad*^−/−^ after *gata1* MO injection at 36 hpf (Fig. S3H), implying that the blood IOL was induced by heme-deficient RBCs. Next, we evaluated HSPC development in *gata1* MO-injected *alas2*^−/−^ or *alad*^−/−^ by WISH and confocal imaging. It showed that the HSPC defects were efficiently restored after blocking the production of heme-deficient RBCs (Fig. S3I-L). Taken together, these results demonstrated that blood IOL was induced by heme-deficient primitive RBCs through the iron exporter Slc40a1, thus leading to the HSPC defects in a non-cell autonomous manner.

### Tfr1b mediates HSPC ferroptosis under blood IOL condition

**Fig. 4.**
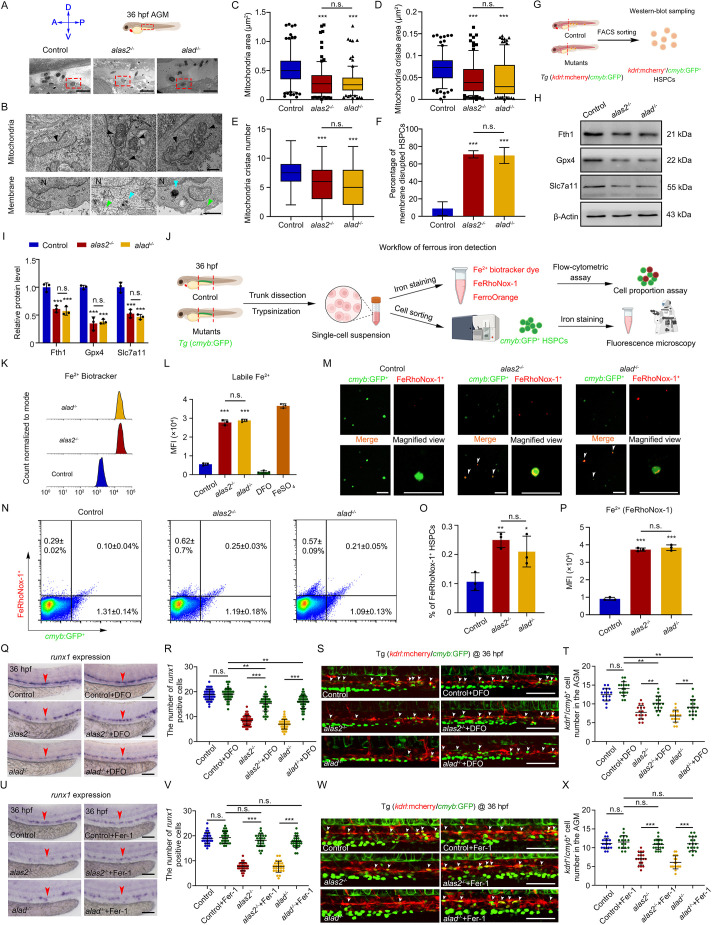
**Iron-dependent HSPC ferroptosis in *alas2*^−/−^ and *alad*^−/−^.** (A) Transmission electron microscopy (TEM) view of a longitudinal section through the artery and vein in control, *alas2*^−/−^ and *alad*^−/−^ AGM region at 36 hpf. (B) TEM view of HSPC subcellular structures. From top to bottom panels are mitochondria and membrane. Arrowheads in black denote mitochondria, green denote plasma membrane and blue denote double-membrane vesicle. (C-E) Quantification of mitochondria area, mitochondria cristae area and cristae number in HSPCs of control, *alas2*^−/−^ and *alad*^−/−^. Box plots show median values (middle bars) and first to third interquartile ranges (boxes); whiskers indicate 1.5 times the interquartile ranges (IQR); individual points from the 10 to 90 percentile are plotted and shown. *n* (mitochondria)=96. (F) Quantification of membrane-disrupted HSPCs in control, *alas2*^−/−^ and *alad*^−/−^. *n* (HSPCs)=12. (G,H) Western-blotting detection of the protein level of Fth1, Gpx4 and Slc7a11 in flow cytometric-sorted HSPCs of control, *alas2*^−/−^ and *alad*^−/−^ at 36 hpf, respectively (30,000 HSPCs were sorted in each group). (I) Quantification of protein levels in H. Protein levels were analyzed using 8-bit-gray analysis (Gel-Pro analyzer). *n*=3 experimental replicates. (J) Schematic workflow for the ferrous iron assessment in HSPCs (*cmyb*^+^) of *alas2*^−/−^ and *alad*^−/−^ with fluorescent iron probes (Fe^2+^ biotracker dye, FeRhoNox-1 and FerroOrange) at 36 hpf. (K) Representative flow cytometric histogram of the Fe^2+^ level in sorted HSPCs of control, *alas2*^−/−^ and *alad*^−/−^ at 36 hpf measured by Fe^2+^ biotracker dye. (L) Quantification of mean fluorescence intensity (MFI) of labile Fe^2+^ level in K. (M) Representative fluorescence images show the co-localization of *cmyb*:GFP^+^ and FeRhoNox-1^+^ cells in *alas2*^−/−^ and *alad*^−/−^ at 36 hpf. The *cmyb*^+^/FeRhoNox-1^+^ cells are denoted by white arrowheads. (N) Flow cytometric analysis of the percentage of *cmyb*^+^/FeRhoNox-1^+^ cells in control, *alas2*^−/−^ and *alad*^−/−^ at 36 hpf. (O) Statistical analysis of the percentage of FeRhoNox-1^+^ HSPCs in N. *n*=3 experimental replicates. (P) Quantification of MFI of Fe^2+^ level in N. *n*=3 experimental replicates. (Q) Expression of HSPC marker *runx1* in control, *alas2*^−/−^ and *alad*^−/−^ with or without DFO treatment (100 μM) at 36 hpf examined by WISH. The AGM regions for marker gene-positive cell counting are denoted by red arrowheads. (R) Quantification of the *runx1*-positive HSPCs in Q. *n*=3 experimental replicates. (S) Confocal imaging shows the *kdrl*^+^/*cmyb*^+^ HSPCs in control, *alas2*^−/−^ and *alad*^−/−^ with or without DFO treatment at 36 hpf. (T) Quantification of the HSPCs in S. *n*=3 experimental replicates. (U) Expression of HSPC marker *runx1* in control, *alas2*^−/−^ and *alad*^−/−^ with or without Ferrostatin-1 (Fer-1) treatment (10 μM) at 36 hpf examined by WISH. The AGM regions for marker gene-positive cell counting are denoted by red arrowheads. (V) Quantification of the *runx1*-positive HSPCs in U. *n*=3 experimental replicates. (W) Confocal imaging shows the *kdrl*^+^/*cmyb*^+^ HSPCs in control, *alas2*^−/−^ and *alad*^−/−^ with or without Fer-1 treatment at 36 hpf. (X) Quantification of the HSPCs in W. *n*=3 experimental replicates. Number of samples are indicated. Data are mean±s.d. *P<0.05, **P<0.01, ***P<0.001 [one-way ANOVA, Tukey's multiple comparisons (C-F,L,O,P,R,T,V,X); two-way ANOVA, Sidak's multiple comparisons (I)]. n.s., not significant. Scale bars: 10 μm (A); 0.5 μm (B); 50 μm (M); 100 μm (Q,S,U,W).

To test whether the decreased number of HSPCs was related to cell apoptosis, we performed TUNEL staining in *alas2*^−/−^ or *alad*^−/−^ under a *Tg* (*fli1a*:GFP) background at 36 hpf. The results showed that the apoptotic signaling was not evident in the AGM region of *alas2*^−/−^ or *alad*^−/−^ (Fig. S4A). Given that HSPC defects occurred under the blood IOL condition, we speculated that the decreased HSPC number might be associated with an iron-dependent cell death, i.e., ferroptosis. Morphologically, ferroptotic cells usually exhibit changes of mitochondria and cell membranes (Dixon et al., 2012; Xie et al., 2016; Yang and Stockwell, 2016). To test this idea, we used transmission electron microscopy (TEM) to examine the morphological changes of HSPCs in control, *alas2*^−/−^ and *alad*^−/−^ at 36 hpf (Fig. 4A). The results showed that HSPCs in both mutants displayed shrunken mitochondria with crista hugging, cytoplasmic and organelle swelling, and plasma membrane rupture, as well as the formation of double-membrane vesicles in the plasma (Fig. 4B-F). However, no ferroptotic features were observed in muscle cells, endothelial cells (ECs) and iron-enriched primitive RBCs (Fig. S4B). Subsequently, we detected the expression of several genes encoding well-established ferroptosis activators in flow cytometry-sorted *kdrl*^+^/*cmyb*^+^ HSPCs or *gata1*^+^ RBCs of control, *alas2*^−/−^ and *alad*^−/−^ at 36 hpf (Fig. S4C). These genes were chosen from a ferroptosis database, FerrDb (www.zhounan.org/ferrdb/current/), including *alox5* (*alox5a*), *alox12*, *acsl4a*, *nox1* and *ptgs2* (*ptgs2a*) (Chen et al., 2021; Zhou and Bao, 2020). The results showed that several lipoxygenase genes (*alox5*, *alox12* and *acsl4a*) were significantly upregulated in the HSPCs of *alas2*^−/−^ or *alad*^−/−^, although normally expressed in the RBCs (Fig. S4D,E). In addition, to further delineate the underlying mechanisms of HSPC ferroptosis, we examined several key ferroptosis suppressors at protein level, including Fth1, Gpx4 and Slc7a11. The results showed that the protein level of these key ferroptosis suppressors was all profoundly reduced in the HSPCs of *alas2*^−/−^ or *alad*^−/−^ (Fig. 4G-I). These results suggested that the ferroptosis processes were activated in the HSPCs of *alas2*^−/−^ or *alad*^−/−^.

To test whether the HSPC ferroptosis was induced by iron-overload, especially the redox-active ferrous iron (Fe^2+^), we used several fluorescent ferrous iron probes to evaluate the HSPC ferrous iron level in *alas2*^−/−^ or *alad*^−/−^ at 36 hpf (Fig. 4J). We first detected that the labile Fe^2+^ level was significantly increased in the HSPCs of *alas2*^−/−^ or *alad*^−/−^ at 36 hpf (Fig. 4K,L). Then, we found that the number of FeRhoNox-1^+^ cells was remarkably increased in the VDA region of control, *alas2*^−/−^ and *alad*^−/−^ by WISH staining with FeRhoNox-1 under zebrafish *Tg* (*fli1a*:GFP) background at 36 hpf (Fig. S4F,G). Subsequently, fluorescence microscopy and flow-cytometric assay showed that the percentage of Fe^2+^-enriched HSPCs and the HSPC Fe^2+^ level were significantly increased in *alas2*^−/−^ or *alad*^−/−^, respectively (Fig. 4M-P). Consistently, FerroOrange staining showed the similar changes in the HSPCs of *alas2*^−/−^ or *alad*^−/−^ (Fig. S4H-J). These results indicated that HSPCs were ferrous iron-overloaded under hematopoietic stress condition.

Next, we used two anti-ferroptosis reagents, Deferoxamine (DFO) and 2,2′-bipyridine (2BP), acting as cell permeable iron chelators, to remove excessive iron in *alas2*^−/−^ or *alad*^−/−^ from 26-36 hpf. After iron chelation, we found that the number of iron-accumulated RBCs was significantly decreased in *alas2*^−/−^ or *alad*^−/−^ at 36 hpf (Fig. S4K,L). At the same time, WISH and confocal imaging showed that the HSPC defects were efficiently restored (Fig. 4Q-T). Consistently, treatment with 2BP also resulted in a partial recovery of HSPC number in *alas2*^−/−^ or *alad*^−/−^ at 36 hpf (Fig. S4M,N). Furthermore, another anti-ferroptosis reagent, Ferrostatin-1, could be also used to rescue HSPC defects in the *alas2*^−/−^ or *alad*^−/−^ at 36 hpf (Fig. 4U-X). Together, these results indicate that the HSPC ferroptosis occurred in *alas2*^−/−^ or *alad*^−/−^, and the discrepancy between different cell types may be due to their distinct sensitivity to iron and activation of anti-ferroptosis systems (Stockwell, 2022).

To investigate how HSPCs responded to excessive iron in *alas2*^−/−^ and *alad*^−/−^, we examined the transferrin receptor family, which is required for cellular iron absorption by transferrin binding and subsequent endocytosis (Gammella et al., 2017; Richard and Verdier, 2020). Recent studies have shown that TfR1 (also known as CD71) level is positively correlated with the iron demand in HSPCs for proliferation and differentiation (Garcia-Prat et al., 2021; Wang et al., 2020). In zebrafish, there are three homologous genes in transferrin receptors: *tfr1a*, *tfr1b* and *tfr2*. Based on the expression patterns, we found that *tfr1b* was specifically enriched in the AGM region at 36 hpf (Fig. S4O), and we also confirmed its specific expression in sorted *kdrl*^+^/*cmyb*^+^ HSPCs by qRT-PCR, whereas *tfr1a* and *tfr2* were barely expressed in HSPCs at 36 hpf (Fig. S4P). Interestingly, we found that both the mRNA level of *tfr1b* in the AGM regions and the protein level in the flow-cytometric sorted HSPCs were moderately upregulated in the *alas2*^−/−^ or *alad*^−/−^ at 36 hpf, respectively (Fig. S4Q-T). Moreover, the *tfr1b* knockdown could efficiently reverse the Fe^2+^ overloaded phenotype in the HSPCs of *alas2*^−/−^ or *alad*^−/−^ (Fig. S4U,V), and restore the HSPC defects (Fig. S4W,X). Therefore, it is plausible that the upregulation of *tfr1b* mediates the excessive iron absorption in HSPCs under IOL condition.

### ROS level is elevated in the AGM region of *alas2* or *alad* mutants

**Fig. 5.**
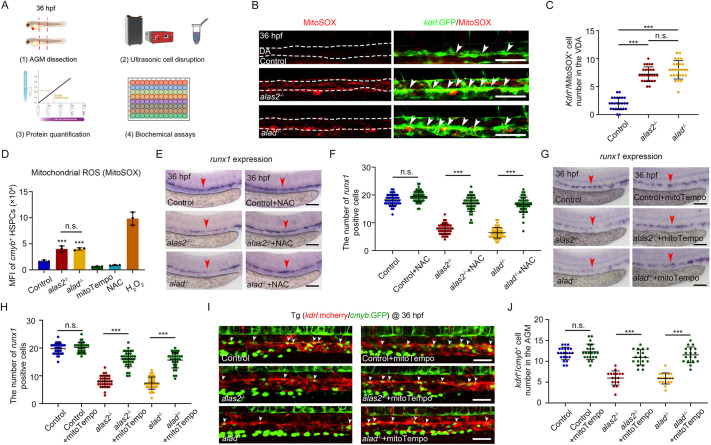
**The HSPC ROS level is elevated in *alas2* and *alad* mutants.** (A) Schematic of biochemical assay of oxidative stress biomarkers (ROS, CAT, SOD, GSH and MDA) in zebrafish dissected trunk regions at 36 hpf. (B) Confocal imaging showing the co-localization of *kdrl*:GFP^+^ and MitoSOX^+^ cells in the VDA of control, *alas2*^−/−^ and *alad*^−/−^ at 36 hpf. The dorsal aorta (DA) regions are denoted by white dashed lines, and the *kdrl*^+^/MitoSOX^+^ cells are denoted by white arrowheads. (C) Quantification of *kdrl*^+^/MitoSOX^+^ cells in B. *n*=3 experimental replicates. (D) Quantification of mean fluorescence intensity (MFI) of mitochondrial ROS level in HSPCs of control, *alas2*^−/−^ and *alad*^−/−^ measured by MitoSOX staining. *n*=3 experimental replicates. (E) Expression of the HSPC marker *runx1* in control, *alas2*^−/−^ and *alad*^−/−^ with or without NAC treatment at 36 hpf examined by WISH. The AGM regions for marker gene-positive cell counting are denoted by red arrowheads. (F) Quantification of the *runx1*-positive HSPCs in E. *n*=3 experimental replicates. (G) Expression of the HSPC marker *runx1* in control, *alas2*^−/−^ and *alad*^−/−^ with or without mitoTempo treatment at 36 hpf examined by WISH. The AGM regions for marker gene-positive cell counting are denoted by red arrowheads. (H) Quantification of the *runx1*-positive HSPCs in G. *n*=3 experimental replicates. (I) Confocal imaging shows the *kdrl*^+^/*cmyb*^+^ HSPCs in control, *alas2*^−/−^ and *alad*^−/−^ with or without mitoTempo treatment at 36 hpf. (J) Quantification of the HSPCs in I.; *n*=3 experimental replicates. Number of samples are indicated. Data are mean±s.d. ***P<0.001 (one-way ANOVA, Tukey's multiple comparisons in C,D,F,H,J). n.s., not significant. Scale bars: 100 μm.

Free ferrous iron causes cellular oxidative stress by increasing reactive oxygen species (ROS) and thus leads to ferroptosis (Ying et al., 2021; Zhou et al., 2018). To test whether the HSPC ferroptosis was induced by iron-related oxidative stress, we evaluated the ROS level and oxidative state in the AGM region of *alas2*^−/−^ or *alad*^−/−^ using biochemical assay kits (Fig. 5A). First, we determined the increased ROS level using DCFH-DA staining in *alas2*^−/−^ and *alad*^−/−^, which was unaltered in embryos with loss of primitive RBCs at 36 hpf (Fig. S5A). Subsequently, we analyzed the anti-oxidative biomarkers, including reduced glutathione (GSH), catalase (CAT) and superoxide dismutase (SOD), and the product of lipid peroxidation, malondialdehyde (MDA) (Fang et al., 2019). It showed that the content of GSH was significantly reduced, and the relative activity of CAT and SOD was decreased, whereas MDA content was significantly increased in the AGM region of *alas2*^−/−^ or *alad*^−/−^ at 36 hpf (Fig. S5B-E). A hallmark of ferroptosis is the accumulation of MDA, and MDA content is one of the indexes to represent the lipid peroxidation level (Lei et al., 2020; Tang et al., 2021). These results implied that the HSPCs were subjected to a higher oxidative stress in *alas2*^−/−^ and *alad*^−/−^.

Moreover, to specify the ROS production in the AGM region, we performed CellROX and MitoSOX staining in *alas2*^−/−^ and *alad*^−/−^ under *Tg* (*kdrl*:mCherry) or *Tg* (*kdrl*:GFP) background, respectively. We observed that the number of *kdrl*^+^/MitoSOX^+^ cells was significantly increased in the VDA of *alas2*^−/−^ or *alad*^−/−^ at 36 hpf (Fig. 5B,C), which was consistent with the CellROX staining results (Fig. S5F,G). Subsequently, we measured the ROS level in the HSPCs of *alas2*^−/−^ or *alad*^−/−^ at 36 hpf by MFI analyses with CellROX and MitoSOX staining. The results showed that the ROS level was increased in HSPCs of both mutants (Fig. 5D; Fig. S5H), suggesting that a higher ROS level was generated in the HSPCs.

To determine whether the ROS generation was iron dependent, we measured the ROS levels in *alas2*^−/−^ or *alad*^−/−^ with or without DFO treatment at 36 hpf. Interestingly, iron chelation could efficiently inhibit the ROS production (Fig. S5I). Next, to test whether the HSPC defects were ROS dependent, we used two potent ROS scavengers, N-acetyl cysteine (NAC) and mitoTempo, to attenuate endogenous ROS in the *alas2*^−/−^ or *alad*^−/−^. NAC and mitoTempo treatments significantly decreased ROS level in the AGM of *alas2*^−/−^ or *alad*^−/−^ at 36 hpf (Fig. S5J,K), and the HSPC defects were also restored efficiently (Fig. 5E-J). Collectively, we concluded that iron-dependent ROS elevation in the AGM region was responsible for the HSPC ferroptosis in *alas2*^−/−^ or *alad*^−/−^.

### Iron-induced ROS causes lipid peroxidation in the AGM region of *alas2*- or *alad-*deficient embryos

**Fig. 6.**
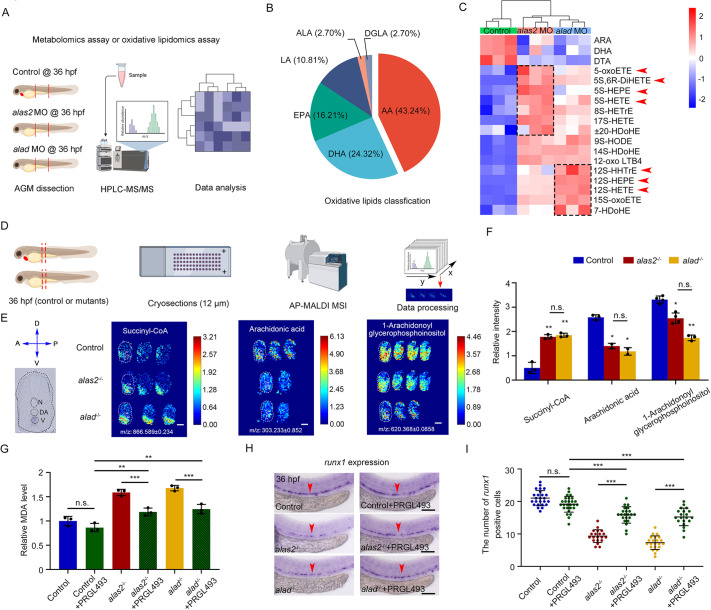
**Iron-mediated AA oxidation leads to overproduction of 5S- and 12S-related ferroptotic stimulus.** (A) Schematic workflow for the metabolomics analyses using the dissected trunk region of control, *alas2*- and *alad*-morphants at 36 hpf. (B) Pie-chart of the proportion of each lipid species in total oxidative lipids. AA, arachidonic acid; ALA, α-linoleic acid; DGLA, dihomo-γ- linoleic acid; DHA, docosahexaenoic acid; EPA, eicosapentaenoic acid; LA, linoleic acid. (C) Heatmaps of differentially regulated oxidative lipids in *alas2* and *alad* morphants. 5S- and 12S-related oxidative lipids are denoted by red arrowheads. (D) Schematic workflow for the MALDI-MSI experiment using control, *alas2*^−/−^ and *alad*^−/−^ at 36 hpf. Cross-cryosections of AGM region are collected (denoted by red dashed rectangle). (E) High resolution MALDI-MS images of succinyl-CoA, AA and 1-arachidonoyl glycerophosphoinositol in control, *alas2*^−/−^ and *alad*^−/−^ at 36 hpf. Left panel is the navigator for cross-section. Relative abundance (mass spectrum peak intensity) of each metabolite is labeled as per the color bars. (F) Statistical analyses of the relative abundance of succinyl-CoA, AA and 1-arachidonoyl glycerophosphoinositol in the VDA regions in H. *n*=2-4 experimental replicates. (G) Relative MDA content in control, *alas2*^−/−^ and *alad*^−/−^ with or without PRGL493 treatment at 36 hpf. PRGL493 treatment from 26 to 36 hpf. *n*=3 experimental replicates. (H) Expression of the HSPC marker *runx1* in control, *alas2*^−/−^ and *alad*^−/−^ with or without PRGL493 treatment at 36 hpf examined by WISH. The AGM regions for marker gene-positive cell counting are denoted by red arrowheads. (I) Quantification of the *runx1*-positive HSPCs in H. *n*=3 experimental replicates. Number of samples are indicated. Data are mean±s.d. *P<0.05, **P<0.01, ***P<0.001 (one-way ANOVA, Tukey's multiple comparisons). n.s., not significant. Scale bars: 100 μm.

Lipid peroxidation is an important type of ROS-induced damage, and excessive oxidative lipids can promote cell ferroptosis (Kinchen et al., 2018; Weismann et al., 2011). We found that genes coding for lipoxygenases, such as, *alox5*, *alox12* and *acsl4a*, were significantly upregulated in the HSPCs of *alas2*^−/−^ or *alad*^−/−^ (Fig. 4D), and the increased MDA content was also detected in *alas2*^−/−^ or *alad*^−/−^ (Fig. S5E). Therefore, to examine the alteration of lipid species in *alas2*- or *alad*-deficient embryos, we performed non-targeted metabolomics assay and targeted oxidative lipidomics assay with dissected trunk region of control, *alas2*- or *alad*-morphants at 36 hpf using ultra high-performance liquid chromatography-mass spectrometry (UHPLC-MS/MS) (Fig. 6A). A total of 476 and 209 differentially regulated metabolites (DRMs) were identified in *alas2*- or *alad*-morphants, respectively, 175 of which were overlapping DRMs (Table S2). Among them, upregulated overlapping DRMs were primarily enriched in the biosynthesis of unsaturated fatty acid pathways, whereas downregulated DRMs were enriched in arachidonic acid (AA) metabolism pathways (Fig. S6A,B). The heatmap showed that the AA and its downstream metabolites were evidently reduced, including prostaglandin G2, prostaglandin D2, 9-HETE, 12-HETE, 8-HEPE and others. (Fig. S6C). Subsequently, we found that 43% (16/37) overlapping upregulated (25) and downregulated (12) oxidative lipids were derived from AA peroxidation in *alas2*- or *alad*-morphants (Fig. 6B; Table S3). Among these elevated AA-derived oxidative lipids, we found that 5S-related (e.g. 5S-HEPE, 5S-HETE and 5S-oxoETE) and 12S-related (e.g. 12S-HEPE, 12S-HHTrE and 12S-HETE) oxidative lipids were highly enriched in *alas2*- or *alad*-morphants, respectively (Fig. 6C), which was consistent with the reduction in AA-related metabolites.

To investigate the alterations of DRMs *in situ*, we used the atmospheric pressure matrix-assisted laser desorption/ionization mass spectrum imaging (AP-MALDI MSI) technique with cross-sections of the AGM region in *alas2*^−/−^ or *alad*^−/−^ at 36 hpf (Fig. 6D). Three out of 14 overlapping DRMs were mapped back to the metabolomic data in *alas2*- or *alad*-morphants, and we found that the AA and 1-arachidonoyl glycophosphoinositol were significantly decreased, whereas the lipid peroxidative precursor Succinyl-CoA was increased in the AGM regions of *alas2*^−/−^ or *alad*^−/−^ (Fig. 6E,F). Together, these results indicate that lipids involved in AA metabolism pathway were altered in the AGM region of *alas2*- or *alad*-deficient embryos.

To further study the triggers in lipid peroxidation, we evaluated the lipoxygenase-based enzymatic reaction and ROS-based Fenton reaction. As Acsl4 has been reported to play an important role in shaping cellular oxidative lipid composition and triggering ferroptosis (Doll et al., 2017; Yang et al., 2016), we employed the Acsl4 inhibitor PRGL493 (Castillo et al., 2021). After the PRGL493 treatment, we found that the MDA content was decreased in the trunk region of *alas2*^−/−^ or *alad*^−/−^ at 36 hpf (Fig. 6G), and the HSPC defects were partially rescued (Fig. 6H,I). These results suggested that the Acsl4-mediated enzymatic reaction was likely responsible for the production of polyunsaturated fatty acid-containing lipid, further leading to the accumulation of oxidative lipids. Next, to investigate the ROS-based Fenton reaction in oxidative lipid production, we performed correlation analysis between iron and oxidative lipids. The results showed that the production of the majority of oxidative lipids was positively correlated to iron, especially the 5S- and 12S-related oxidative lipids in *alas2*- or *alad*-morphants (Fig. S6D,E). Subsequently, we measured the MDA content upon abnormal RBC elimination (*gata1* MO injection), iron chelation (DFO treatment) and ROS attenuation (NAC treatment) (Fig. S6F). Interestingly, all the treatments efficiently reduced the MDA content (Fig. S6G-I), implying that the Fenton reaction was a major factor in lipid peroxidation, which resulted from excessive iron-induced ROS elevation by heme-deficient-RBCs.

### The formation of erythrocyte-biased HSPCs is impaired in *alas2*- or *alad*-deficient embryos

**Fig. 7.**
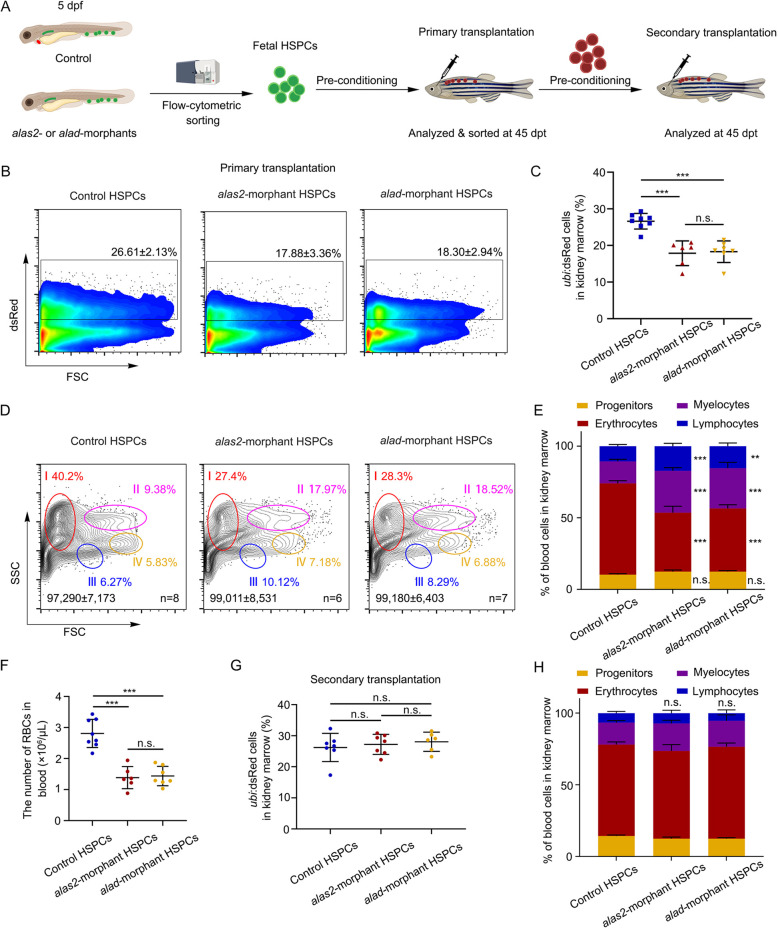
**HSPCs derived from *alas2*- and *alad*-deficient embryos show low erythroid lineage reconstitution efficiency.** (A) Schematic workflow for the primary and secondary HSPC transplantation with sorted *ubi*:dsRed^+^/*CD41*:GFP^low^ HSPCs in *alas2* and *alad* morphants at 5 dpf. (B) Flow cytometric analysis of engrafted *ubi*-dsRed^+^ cells in the recipient kidney marrow (KM) at 45 dpt. Number of recipients: *n* (control HSPCs>irradiated WT)=8, *n* (*alas2*-morphant HSPCs>irradiated WT)=6, *n* (*alad*-morphant HSPCs>irradiated WT)=7. (C) Statistical analysis of the engraftment efficiency in B; the total numbers of recipients are indicated. (D) Flow cytometric analysis of different blood cell types in recipient kidney marrows. Each circle and percentile represent different cell lineages (I, erythrocytes; II, myelocytes; III, lymphocytes; IV, progenitors). (E) Quantification of different blood lineages in the kidney marrows based on D. Total cell numbers in whole kidney marrows are indicated. (F) Statistical analysis of the number of RBCs in recipient peripheral blood. The total numbers of recipients are indicated. (G) Statistical analysis of the engraftment efficiency of control, *alas2* and *alad* morphants after secondary transplantation. Number of recipients: *n* (control HSPCs>irradiated WT)=7, *n* (*alas2*-morphant HSPCs>irradiated WT)=7, *n* (*alad*-morphant HSPCs>irradiated WT)=5. Total numbers of recipients are indicated. (H) Quantification of different blood lineages in the kidney marrows based on G. Total numbers of recipients are indicated. Number of samples are indicated. Data are mean±s.d. **P<0.01, ***P<0.001 [one-way ANOVA, Tukey's multiple comparisons (C,F,G); two-way ANOVA, Sidak's multiple comparisons (E,H)]. n.s., not significant.

To characterize the functional role of impaired HSPC subpopulations, we performed WISH to detect the multilineage differentiation of HSPCs in the *alas2*^−/−^ or *alad*^−/−^ at 5 dpf. The results showed that the myeloid [*pu.1* (*spi1b*), *l-plastin* and *lyz*] and lymphoid (*rag1*) markers were normally expressed, whereas the expression of HSPC (*cmyb*) and erythroid (*gata1*) markers was significantly decreased (Fig. S7A,B). To further confirm this finding, we performed primary and secondary HSPC transplantation with flow cytometry-sorted *ubi*:dsRed^+^/*CD41*:GFP^low^ HSPCs in control, *alas2*- or *alad*-morphants at 5 dpf injecting into allogenic irradiated 3-month-old wild-type adult zebrafish (Fig. 7A). After 45 day post transplantation (dpt), we detected that the proportion of *alas2*- or *alad*-morphant HSPC-derived *ubi*:dsRed^+^ cells was significantly lower than that in control groups (Fig. 7B,C). Then, the multilineage analyses showed that the erythroid lineage reconstitution efficiency of *alas2*- or *alad*-morphant-derived HSPCs was decreased (Fig. 7D,E). At the same time, we detected that the number of circulating RBCs was decreased in the peripheral blood of recipients transplanted with *alas2*- or *alad*-morphant HSPCs (Fig. 7F). Next, to test whether the multilineage repopulating hematopoietic stem cells (HSCs) could survive and self-renew in *alas2*- or *alad*-morphants, we performed secondary transplantation with primary recipient-derived *ubi*:dsRed^+^/*CD41*:GFP^low^ HSPCs (Fig. 7A). At 45 dpt, both control and morphant-derived HSPCs displayed similar reconstitution efficiencies and equivalent multilineage repopulating potential (Fig. 7G,H). These results support a model that erythrocyte-biased HSPCs undergo ferroptosis in heme-deficient primitive RBC-induced iron overload stress conditions (Fig. S7C). Taken together, these data suggest that in *alas2*- or *alad*-deficient embryos, the ferroptotic HSPCs were most likely erythrocyte-biased, whereas the generation of multilineage repopulating HSCs was not affected.

## DISCUSSION

The timeline of hematopoietic cell generation is largely conserved during vertebrate embryogenesis, as reflected in the primitive, pro-definitive and definitive waves. It is proposed that the AGM microenvironment established by primitive hematopoietic cells is important for HSPC generation and maintenance. Here, for the first time, our study revealed that healthy primitive RBCs are essential for HSPC formation, evidenced by the fact that heme-deficient primitive RBC-induced IOL stress leads to definitive HSPC ferroptosis. This new finding should contribute to our understanding of the functional niche in definitive HSPC generation, and also provide insights into the *in vitro* HSC induction.

### Primitive RBCs in regulating iron homeostasis during embryogenesis

In early embryogenesis, circulating primitive RBCs in the blood are crucial for maintaining iron homeostasis during early embryogenesis, and transferrin α is required for iron transport from the yolk to the embryo in zebrafish (Fraenkel et al., 2009; Ganz and Nemeth, 2012). A substantial fraction of iron is used for heme biosynthesis during erythropoiesis, and the impairment in the heme synthesis pathway results in iron accumulation in RBCs due to an incorrect demand signal for iron requirements. Our findings suggest that the heme biosynthesis pathway is important for primitive RBC iron hemostasis and trafficking. In the absence of primitive RBCs, the blood iron level is slightly decreased (Fig. S3F), implying that the yolk iron cannot be efficiently used, and other cell types play a complementary role in maintaining iron homeostasis. The iron transporter Slc40a1 is the only known iron exporter in vertebrates and is essential for cellular iron homeostasis (Hentze et al., 2010). Consistently, the expression of *slc40a1* in erythroblasts has been previously identified in our mouse fetal liver single-cell RNA-seq database (http://liulab.ioz.ac.cn/fetal_liver), and also in a recent RBC-related single-cell RNA-seq study (Gao et al., 2022; Xu et al., 2022). Previous studies have shown that mutations in SLC40A1 impair hepcidin-ferroportin binding and thus cause iron-overload syndrome in macrophages and hepatocytes (Brissot et al., 2018; Mayr et al., 2011). Similarly, we found that Slc40a1 is responsible for exporting excessive iron in RBCs, and both abnormal RBC elimination and Slc40a1 inhibition attenuated the blood iron level (Fig. 3G). These findings demonstrate that the heme-deficient primitive RBC is responsible for triggering the IOL stress condition during early embryogenesis and that Slc40a1 acts as a mediator for iron export.

### The regulation of HSPC ferroptosis by primitive RBCs

Considering that iron is necessary for HSC induction *in vitro*, it is plausible that a suitable iron level can promote HSC induction and reconstitution (Zhang et al., 2022). However, a recent study demonstrated that human HSC is sensitive to ferroptosis under blood disease conditions, due to the absence of the histone deubiquitinase MYSM1 (Zhao et al., 2023a). Consistently, we found that excessive iron induces HSPC ferroptosis under IOL stress condition in *alas2*- and *alad*-deficient embryos. The Transferrin receptor plays a crucial role in responding to extracellular iron, and is tightly controlled by several cellular signal pathways. The upregulation of transferrin receptor has been regarded as a feature of iron absorption, including the inhibition of the TFEB-mediated lysosomal degradation pathway in HSPCs (Garcia-Prat et al., 2021) and the activation of several iron regulatory protein (IRP) dependent pathways (Jiang et al., 2014; Martelli et al., 2015). In zebrafish, there are three homologous transferrin receptor genes: *tfr1a*, which is specifically expressed in the developing erythroid precursors (Wingert et al., 2004); *tfr1b*, which is enriched in VDA (in this study); and *tfr2*, which is primarily expressed in hepatocytes and erythroid cells. As a result of dysregulated iron uptake, the expression of *tfr1b* is upregulated in the HSPCs of *alas2*^−/−^ or *alad*^−/−^ (Fig. S4K).

Different from RBCs or other cell types, excessive iron in HSPCs directly leads to ferroptosis, implying that there may exist distinct responses to excessive iron across different cell types. Accordingly, we hypothesized that RBCs might not be sensitive to the IOL condition because of their anti-ferroptotic gene expression patterns, such as upregulation of certain anti-ferroptotic genes (*fth1a*, *slc11a2*) and downregulation of activators (*slc3a2b*, *alox5a*, *alox5ap*) (Fig. 3G). In mammals, TFR1 has been identified to be a specific ferroptosis marker that regulates ferroptosis progression (Feng et al., 2020). Therefore, the upregulation of *tfr1b* may mediate the HSPC ferroptosis in the context of heme-deficient primitive RBC conditions. Here, we show that zebrafish *alas2*^−/−^ and *alad*^−/−^ can serve as IOL disease models, which are similar to mouse models (Abraham et al., 1985; Chiabrando et al., 2014). Our findings here provide an important resource to manipulate the ferroptosis process that is instructive for clinical therapies of IOL-related disease.

Our work also demonstrates that ferroptotic HSPCs are erythrocyte-biased subpopulations, the loss of which results in decreased erythroid lineage reconstitution efficiency. In fact, Tfr1-positive HSPCs have been characterized with erythroid differentiation potential (Garcia-Prat et al., 2021; Wang et al., 2020; Zhang et al., 2022). Intriguingly, secondary transplantation assays suggest that the HSPCs with multilineage repopulating potential are not affected in IOL stress condition. A previous study has shown that the degradation of TfR1 is required to maintain HSC quiescence, whereas Tfr1 is enriched in erythrocyte-biased HSPCs and is essential for erythroid lineage differentiation (Garcia-Prat et al., 2021). Our study also suggests that erythrocyte-biased HSPCs with higher transferrin receptor expression are more sensitive to heme-deficiency induced ferroptosis.

In summary, this work reveals that heme-deficient primitive RBCs can induce definitive HSPC ferroptosis via impairment of iron homeostasis of the AGM niche. As a result, the higher local blood iron level is detrimental to HSPC production. Our findings suggest that iron-induced excess ROS production is the main cause of lipid peroxidation, thereby triggering HSPC ferroptosis. Therefore, a heme-deficient primitive RBC-iron-ROS-lipid peroxidation axis plays a crucial role in regulating HSPC ferroptosis under IOL stress conditions.

## MATERIALS AND METHODS

### Zebrafish lines

Zebrafish strains were maintained on a 14 h light /10 h dark cycle in system water (conductivity 500-550 μs/cm, pH 7.0-7.5, dissolved oxygen≥6.0 mg/l) at 28.0±0.5°C (mean±s.d.) under standard conditions. Strains including Tübingen (Tü), *Tg* (kdrl:mCherry) (Bertrand et al., 2010), *Tg* (*kdrl*:GFP) (Jin et al., 2005), *Tg* (*fli1a*:GFP) (Lawson and Weinstein, 2002), *Tg* (*ubi*:dsRed/*CD41*:GFP) (Lv et al., 2020), *Tg* (*cmyb*:GFP) (North et al., 2007), *Tg* (*gata1*:dsRed) (Traver et al., 2003), *Tg* (*gata1*:dsRed/*kdrl*:GFP) (Jin et al., 2007), and zebrafish mutants *alas2*^−/−^ and *alad*^−/−^ were previously generated in our laboratory and were maintained as a heterozygous breeding colony. All zebrafish mutants were maintained on a Tü background and siblings were used for all experiments involving the *alas2* and *alad* lines. The zebrafish embryos were obtained by natural spawning of adult males and females. All zebrafish experiments in this study were approved by the Ethical Review Committee of the Institute of Zoology, Chinese Academy of Sciences, China.

### Morpholino microinjection and mutant generation

The antisense MOs, including *tif1γ* , *gata1* , *klf1* , *klf3* , *alas2* , *alad* and *tfr1b* Mos, were purchased from GeneTools. Stock solutions of 1 mM MO were prepared by ddH_2_O dilution, and 1-4 ng of MOs were injected into one-cell-stage zebrafish embryos. All MO sequences are shown in Table S4.

The *alas2* and *alad* mutants were generated by using a CRISPR/Cas9 technique with gene-specific guide RNA (gRNA) and Cas9 mRNA. The *alas2* (ENSDARG00000038643) gRNA was designed for targeting exon 3 (target sequence, GGAGGATGTCCAGCCCAATC). The *alad* (ENSDARG00000100372) gRNA was designed for targeting exon 8 (target sequence, CCTGGTGCCCGAGGACTAGC). The syntheses of gRNA and Cas9 mRNA was carried out according to previously published methods (Chang et al., 2013). In detail, the gRNAs were generated using *in vitro* transcription by T7 RNA polymerase, and capped Cas9 mRNA was generated using T7 mMessage Machine Kit (Thermo Fisher Scientific) with linearized pXT7-Cas9 plasmid (a gift from Prof. Jing-Wei Xiong, Peking University, China). Then, gRNA and Cas9 mRNA were purified using RNA clean kit (TIANGEN) and miRNA isolation kit (Ambion), respectively, following the manufacturer's instructions. Mutant genotyping was carried out by DNA-sequencing and the primers used for *alas2* and *alad* mutants genotyping are listed in Table S4.

### Whole-mount *in situ* hybridization

The WISH experiments were performed by using a ZF-A4 *in situ* hybridization machine (Zfand) with digoxigenin-uridine-5′-triphosphate (DIG) labeled single-stranded RNA probes. RNA probes targeting genes of *alas2*, *alad*, *runx1*, *cmyb*, *tfa*, *slc40a1*, *tfr1a*, *tfr1b*, *tfr2*, *rag1*, *pu.1*, *l-plastin*, and *lyz* were synthesized following standard methods with some modifications (Jacobs et al., 2011). The gene-specific PCR products were firstly cloned into pGEM-T vector (Promega), and then transcribed by T7 or SP6 RNA polymerase (Promega). Primers used for RNA probe generation are listed in Table S4. WISH procedures were followed as previously described (Wang et al., 2011).

### O-dianisidine staining, DAB-enhanced Prussian blue staining

Hemoglobin level was detected by O-dianisidine staining at room temperature for 15-30 min in the dark as previously reported (Paffett-Lugassy and Zon, 2005). Dechorionated embryos were stained with working solution containing 0.6 mg/ml O-dianisidine (Sigma-Aldrich), 0.01 M sodium acetate (pH 4.5), 0.65% H_2_O_2_ and 40% (v/v) ethanol.

The 3,3-diaminobenzidine (DAB)-enhanced Prussian Blue iron staining was performed to detect ferric iron in whole zebrafish embryos (Lumsden et al., 2007). In detail, the fixed embryos were immersed in potassium ferrocyanide solution (3%) (ScyTek) containing 2.5% potassium ferrocyanide and 0.25 M HCl for 30 min at room temperature, then rinsed three times in 1× phosphate-buffered saline (PBS) with 0.1% Tween 20 (PBST). Next, the embryos were incubated in 0.3% H_2_O_2_ (dissolved in methanol) for 20 min at room temperature. Following two rinses in 1× PBST, embryos were incubated for 10-15 min in DAB peroxidase substrate (dissolved in 1× PBS) (Sigma-Aldrich). Finally, embryos were rinsed three times in 1× PBST and stored in glycerol for microscopy.

### TUNEL and fluorescent ferrous iron staining

The TUNEL staining was performed as previously reported (Heng et al., 2020). In brief, the zebrafish embryos of control, *alas2*^−/−^ and *alad*^−/−^ under *Tg* (*fli1a*:GFP) background at 36 hpf were fixed in 4% paraformaldehyde (PFA) (Merck) and then dehydrated with methanol at −20°C for more than 2 h. After rehydration, the embryos were washed three times with 1× PBST and treated with 10 μg/ml Proteinase K (Amersco) for permeabilization. The permeabilized embryos were fixed in 4% PFA for 20 min at room temperature. After washing three times with 1× PBST, the embryos were incubated with a TUNEL labeling mixture (*In Situ* Cell Death Detection Kit TMR Red, Roche) at 4°C overnight. After washing with 1×PBST, the embryos were observed under a confocal microscope (A1R, Nikon).

A single-cell suspension was prepared with the trunk region of control, *alas2*^−/−^ and *alad*^−/−^ under *Tg* (*cmyb*:GFP) background, For HSPC ferrous iron level detection, the cells were stained for 1.5 h at 28°C with 5 μM Fe^2+^ biotracker dye (Sigma-Aldrich), or 1 h at 28°C with 5 μM FeRhoNox-1 (RuiTaibio), or 1 h at 28°C with 1 μM FerroOrange (RuiTaibio) and 1 μg/ml Hoechst 33342 (Invitrogen). After staining, cells were washed with 1× PBS and analyzed by flow-cytometry and confocal microscope (A1R, Nikon).

### Total iron quantification

The indicated tissue or cell of the zebrafish embryo was collected for iron quantification using the Iron Assay Kit (colorimetric) (Abcam) or Inductively Coupled Plasma-Mass Spectrometry (ICP-MS) (7800X, Agilent) based on the manufacturer's instructions and as previously reported (Javed et al., 2019). In brief, samples were homogenized using the Bioruptor sonication device (Diagenode). The iron content of body tissue was normalized to weight (∼0.0219-0.0258 g/150 embryos), yolk was homogenized into a certain volume (100 μl/150 embryos), blood sample collection was carried out as previously described with some modification (Van Wijk et al., 2019) and diluted into an optimal volume to meet the detection limit of the iron assay kit (∼0.2 μl/300 embryos, diluted into 40 μl), and the sorted cell sample was normalized to cell number (1×10^6^ cells).

### CellROX and MitoSOX staining

The CellROX^®^ Green Reagent (Thermo Fisher Scientific) and MitoSOX™ Red (Invitrogen) were used to analyze *in situ* cellular ROS level as previously described (Alberto Rissone, 2016). The 36 hpf zebrafish embryos of control, *alas2*^−/−^ and *alad*^−/−^ under *Tg* (*kdrl*:GFP) or *Tg* (*kdrl*:mcherry) background were stained with MitoSOX and CellROX, respectively. After staining, embryos were washed with 1× PBS and observed by fluorescence microscope (Nikon).

### Bright-field and confocal microscopy

The bright-field photographs of WISH, biochemical staining and blood were taken using a stereomicroscope (SMZ1500, Nikon). High-resolution images and movies of blood flow were taken under an upright microscope (Eclipse 80i, Nikon). Fluorescent images were taken using a confocal microscope (A1R+SIM, Nikon). Samples for microscopic observation and photography were prepared as previously described (Renaud et al., 2011). For live imaging, the embryos were anesthetized with 100 μg/ml tricaine, then embedded using 1.2% low melting agarose in a Nunc™ glass bottom dish (15068, Thermo Fisher Scientific).

### Western blotting

The protein level was detected by following the previously described protocol (Lv et al., 2020). In detail, whole embryos of *alas2* or *alad* mutants and their siblings at 36 hpf were collected for Alas2 and Alad protein level detection using anti-Alas2 (1:2000, GTX127887, GeneTex) or anti-Alad (1:2000, ab59013, Abcam) antibody, respectively. The flow cytometric-sorted 30,000 HSPCs (*kdrl*^+^/*cmyb*^+^) of control, *alas2^−/−^* or *alad*^−/−^ at 36 hpf were used for detection of Fth1, Gpx4, Slc7a11 and Tfr1b protein levels using anti-Fth1 (1:2000, A19544, ABclonal), anti-Gpx4 (1:1000, A1933, ABclonal), anti-Slc7a11 (1:1000, A15604, ABclonal) and anti-Tfr1b (1:2000, 10084-2-AP, Proteintech) antibodies, respectively.

### Flow-cytometric cell sorting

Flow cytometric cell sorting was performed as previously reported with a MoFlo XDP (Beckman) (Lv et al., 2020). For EC (*kdrl*:mcherry^+^) and HSPC (*kdrl*:mcherry^+^/*cmyb*:GFP^+^) sorting, the trunk region of 36 hpf embryos (*alas2*^−/−^, *alad*^−/−^ and their siblings) under *Tg* (*kdrl*:mcherry/*cmyb*:GFP) background was dissected using a 2-ml injection syringe and dispersed to the single-cell suspension with 0.5% trypsin at 28°C. Then, the digestion was stopped by adding fetal bovine serum (FBS) up to 10%. The single-cell suspension was filtered through a 300 mesh (50 μm) nylon cell-strainer filtering membrane. For RBC (*gata1*:dsRed^+^) sorting, whole 36 hpf embryos (*alas2*^−/−^, *alad*^−/−^ and their siblings) under *Tg* (*gata1*:dsRed) background were collected. The preparation of single-cell suspension was as described above. All the cell types were sorted based on fluorescent colors and collected into 1× PBS containing 1% FBS.

### RNA extraction and qRT-PCR

For body or tissue total RNA collection, the whole embryo or dissected trunk region of *alas2*^−/−^, *alad*^−/−^ and their siblings were extracted using TRIzol reagent (Life Technologies) following the manufacturer's instructions. For sorted cells, the total RNA of HSPCs (500 cells) was extracted using the RNeasy Micro Kit (Qiagen, 74004). The cDNA was reverse transcribed using M-MLV Reverse Transcriptase (Promega, M1701). The primer sequences used for qRT-PCR are listed in Table S4.

### Bulk RNA-seq and data processing

The cDNA samples of sorted RBCs (*gata1*:dsRed^+^) were sequenced using Illumina NovaSeq 6000. A total of 50,000 RBCs were used per sample for RNA-seq experiments. The quality control of raw sequencing data was performed using FastQC, and low-quality bases were trimmed and filtered by Cutadapt (V 4.1) and Trimmomatic (V 0.32). Then, the reads were mapped to the zebrafish (*Danio rerio*) gene information from the National Center for Biotechnology Information (NCBI) database. The fold change of differentially expressed genes was analyzed using the R package DEGseq2 (V 1.37.5). Gene Ontology (GO) analysis was performed with the online Gene Ontology Resource (http://geneontology.org/).

### Transmission electron microscopy

The trunk region of *alas2*^−/−^, *alad*^−/−^ and their siblings at 36 hpf was fixed with 2.5% (vol/vol) glutaraldehyde and 2% PFA in PBS (0.1 M, pH 7.4). Then, the tissues were immersed in 1% (wt/vol) OsO_4_ and 1.5% (wt/vol) potassium ferricyanide aqueous solution at 4°C for 1 h. After washing with PBS, the tissues were incubated in filtered 1% thiocarbohydrazide aqueous solution (Sigma-Aldrich) at room temperature for 30 min, followed by 1% unbuffered OsO_4_ aqueous solution at 4°C for 1 h and 1% uranyl acetate (UA) aqueous solution at room temperature for 2 h. The tissues were dehydrated through an ethanol series (30%, 50%, 70%, 80%, 90%, 100%, 100%, 10 min each, at 4°C). Then, the tissues were transferred into pure acetone for 10 min (twice). Tissues were infiltrated in graded mixtures of acetone and SPI-PON812 resin (21 ml SPI-PON812, 13 ml DDSA and 11 ml NMA) (3:1, 1:1, 1:3). Finally, the tissues were embedded in pure resin with 1.5% BDMA and polymerized at 45°C for 12 h, followed by at 60°C for 48 h. The ultrathin sections (70 nm thick) were sectioned using a microtome (Leica, EM UC6), and examined using a transmission electron microscope (FEI Tecnai Spirit 120 kV).

### Chemical treatment

For ferroportin inhibitor VIT2763 (MCE) treatment, the optimized concentration (100 nM) was tested in our pre-experiments based on manufacturer's instructions and a previous report (Manolova et al., 2019). For iron chelator treatment, DFO (Sigma-Aldrich) and 2BP (Sigma-Aldrich) were used at 100 μM and 10 μM, respectively (Dou et al., 2019; Elenbaas et al., 2016). For antioxidant treatment, NAC (Sigma-Aldrich) and mitoTempo (Sigma-Aldrich) were dissolved at 100 μM or 10 μM, respectively, as previously described (North et al., 2010; Zhao et al., 2023b). For anti-ferroptosis treatment, the well-established reagent Ferrostatin-1 was used at 10 μM (Zilka et al., 2017). For the ACSL4 inhibitor PRGL493 treatment, a concentration of 100 μM was used.

### Biochemical assays

The dissected trunk region of 36 hpf embryos (control, *alas2*^−/−^ and *alad*^−/−^) was used for analyses of oxidative indicators. In detail, the samples were homogenized on ice with nine volumes of cold PBS using a Bioruptor sonication device (Diagenode). The supernatants of the homogenate were collected for biochemical assays after centrifugation (400 ***g***, 4°C) for 15 min. The 24-well plates (NUNC) were read using a BioTek Cytation5 imaging reader (BioTek). All the commercially available kits were obtained from Nanjing Jiancheng Bioengineering Institute, including the ROS Assay Kit (chemical fluorescence method, DCFH-DA staining), SOD assay kit (hydroxylamine method), CAT assay kit (visible light), GSH assay kit (colorimetric method), MDA assay kit (TBA method) and total protein quantitative assay kit (Coomassie Brilliant Blue method). The measurement of each index in indicated samples was carried out according to the manufacturer's instructions.

### Untargeted metabolomics assay

The sample was freeze dried and extraction solution (acetonitrile:methanol:water=2:2:1) with 1 μg/ml internal standard was added. After 30 s vortex, the samples were homogenized at 35 Hz for 4 min and sonicated for 5 min on ice. The homogenization and sonication cycle were repeated three times. Then the samples were incubated for 1 h at −40°C and centrifuged at 13,800 ***g*** for 15 min at 4°C. The resulting supernatant was transferred to a fresh glass vial for analysis. The quality control sample was prepared by mixing an equal aliquot of the supernatants from all of the samples.

LC-MS/MS analyses were performed using a UHPLC system (1290, Agilent Technologies) with a UPLC HSS T3 column (2.1 mm×100 mm, 1.8 μm) coupled to a Q Exactive mass spectrometer (Orbitrap MS, Thermo Fisher Scientific). The mobile phase A was 0.1% formic acid in water for positive mode, and 5 mmol/l ammonium acetate in water for negative mode, and the mobile phase B was acetonitrile. The elution gradient was set as follows: 0∼1.0 min, 1% B; 1.0∼8.0 min, 1%∼99% B; 8.0∼10.0 min, 99% B; 10.0∼10.1 min, 99%∼1% B; 10.1∼12 min, 1% B. The flow rate was 0.5 ml/min. The injected volume was 2 μl. The QE mass spectrometer was used for its ability to acquire MS/MS spectra on the information-dependent acquisition (IDA) mode in the control of the acquisition software (Xcalibur 4.0.27, Thermo Fisher Scientific). In this mode, the acquisition software continuously evaluates the full scan MS spectrum. The ESI source conditions were set as following: sheath gas flow rate as 45 Arb, Aux gas flow rate as 15 Arb, capillary temperature 400°C, full MS resolution as 70,000, MS/MS resolution as 17,500, collision energy as 20/40/60 in NCE mode, spray voltage as 4.0 kV (positive) or 3.6 kV (negative).

### Oxidative lipidomics assay by UHPLC-MS/MS

For solid samples, a 50 mg aliquot of each individual sample was precisely weighed and transferred to an Eppendorf tube. After the addition of 600 μl of extract solution [80% methanol/water (v/v), precooled to −40°C, containing isotopically-labeled internal standard mixture], the samples were vortexed for 30 s, and homogenized at 35 Hz for 4 min and sonicated for 5 min in the ice-water bath. The homogenate and sonicate cycle was repeated twice, followed by storing the sample at −40°C for 1 h. After centrifugation (15 min, 13,800 ***g***, 4°C), a 480 μl aliquot of the supernatant was transferred to an Eppendorf tube and 320 μl water was added. After vortexing for 30 s, the sample was further purified using solid-phase extraction (SPE). The SPE cartridges were equilibrated with 1 ml of methanol and 1 ml of water. After loading a sample (supernatant obtained following the procedure described above), the cartridge was washed with 1 ml of 5% methanol/water (v/v). The flow-through fraction was then discarded. Finally, the samples were eluted with 1 ml of methanol, and then the eluent was evaporated to dryness under a gentle stream of nitrogen and reconstituted in 100 μl of 30% acetonitrile/water (v/v). The reconstituted solutions were vortexed for 30 s, homogenized at 60 Hz for 4 min and sonicated for 5 min in the ice-water bath. After centrifugation (1 min, 13,800 ***g***, 4°C), the reconstituted solution was transferred to an Eppendorf tube with a filter membrane. After centrifugation (15 min, 13,800 ***g***, 4°C), the clear supernatant was subjected to UHPLC-MS/MS analysis.

The ultra high-performance liquid chromatography separation was carried out using an ExionLC System (Sciex), equipped with a Waters Acquity UPLC BEH C18 column (150×2.1 mm, 1.7 μm, Waters). The mobile phase A was 0.01% formic acid in water, and the mobile phase B was 0.01% formic acid in acetonitrile. The column temperature was set at 50°C. The auto-sampler temperature was set at 4°C and the injection volume was 10 μl. A Sciex 6500 QTRAP^+^ triple quadrupole mass spectrometer (Sciex), equipped with an IonDrive Turbo V electrospray ionization (ESI) interface, was applied for assay development. Typical ion source parameters: Curtain Gas=40 psi, IonSpray Voltage=−4500 V, temperature=500°C, Ion Source Gas 1=30 psi, Ion Source Gas 2=30 psi.

### Cryosections and MALDI-MSI

Upon reaching the 36 hpf stage, the dechorionated zebrafish embryos were embedded in a 10% (w/v) gelatin solution and immediately placed into nitrogen. Then, the embedded embryo was transferred to a cryotome (CM3050S, Leica) and the embryos were cryo-sectioned at 10 µm thickness. Sections were attached to the pre-chilled Indium-Tin-Oxide (ITO)-coated glasses slides (25×75 mm, 1.1 mm thickness, <7 ohm/sq, Kaivo). The prepared slides were placed on a −80°C pre-chilled aluminum block and lyophilized under a vacuum desiccator for ∼1 h. Then, the DHB matrix (20 mg/ml) was applied using a robotic TM-Sprayer™ matrix application system (HTX Technologies), as previously described (Asslan et al., 2021). Next, the prepared tissue sections were dried in a vacuum desiccator for 30 min before imaging. All sections were imaged within 24 h of sectioning.

All matrix-assisted laser desorption/ionization mass spectrometry imaging (MALDI-MSI) data were acquired on a Xevo G2-XS QTOF (Waters) operated by FlexImaging 5.0 software (Bruker Daltonics). The detailed instrumental parameters were used based on those previously reported (Liang et al., 2021), with some modifications. MALDI-MSI was obtained using a smartbeam 3D laser (Bruker Daltonics) at a repetition rate of up to 10,000 Hz in negative ion mode at a spatial resolution of 50 μm. Key acquisition parameters including laser power (75%), mass range (m/z 400-1000), the number of laser shots (1000), detector gain voltage (3.0×2810 V), reflector voltage (20.84 kV), lens voltage (11.00 kV), ion source voltage (20 kV), ion extraction time (100 ns) and the matrix suppression (m/z 320) were optimized and fixed during the whole experiment.

Data were analyzed using SCiLS Lab 2016a software (Bruker Daltonics). Normalization was performed on the basis of the total ion count, and masses were selected with variable mass-selection window widths set at ±0.10 Da. Spatial segmentation and principal component analysis (PCA) were applied for unsupervised analyses. Receiver operating characteristic (ROC) analysis was performed to screen organ-specific lipids and select differential lipids between the control, *alas2*^−/−^ and *alad*^−/−^ zebrafish.

### Zebrafish HSPC transplantation

The HSPC transplantation assays were conducted as previously reported (Fraint et al., 2023; Lv et al., 2020). The zebrafish embryos of control, *alas2*- and *alad*-morphants under *Tg* (*ubi*:dsRed/*CD41*:GFP) background at 5 dpf were HSPC donors, and irradiated adult wild-type zebrafish was used as recipients. The engraftment efficiency of primary and secondary transplantation was analyzed at 45 dpt by MoFlo XDP (Beckman), and the results were processed using FlowJo (10.5.0) and Summit (5.1.0) software.

### Quantification and statistical analysis

Data analysis was performed using Prism software (Version 9, GraphPad Software), and the values used to create all the graphs in the figures are listed in Table S5. Results were expressed as mean±standard deviation (s.d.). Statistical significance was determined using a Mann–Whitney non-parametric *U*-test when the experiment contained two groups, or one-way or two-way ANOVA when comparing more than two groups. Post-hoc analysis was performed on the ANOVA using the Tukey's or Sidak's post hoc tests as described in the figure legends. Before analyzing the statistical significance of differences among treatments, we tested whether the variance was similar using either the *F*-test or Bartlett test. The level of conventional statistical significance was set at *P*<0.05. The statistical parameters can be found in the figures and the figure legends. The relative mean fluorescence intensities were analyzed using ImageJ. For statistical analysis of metabolomics data, the raw data were converted to the mzXML format using ProteoWizard and processed using an in-house program, which was developed using R and based on XCMS, for peak detection, extraction, alignment and integration. Then an in-house MS2 database (BiotreeDB) was applied for metabolite annotation. The cutoff for annotation was set at 0.3. Statistical analysis of oxidative lipidomics data was carried out using Sciex Analyst Work Station Software (Version 1.6.3), and Multiquant 3.03 software was employed for MRM data acquisition and processing.

## Supplementary Material

10.1242/develop.201690_sup1 Supplementary information
