# Correction: Heme-deficient primitive red blood cells induce HSPC ferroptosis by altering iron homeostasis during zebrafish embryogenesis

**DOI:** 10.1242/dev.204839

**Published:** 2025-05-06

**Authors:** Peng Lv, Shuai Gao, Feng Liu

There was an error in Development (2023) **150**, dev201690 (doi:10.1242/dev.201690).

Shuai Gao was not included in the author list. The correct author list appears above.

The Author contributions list has also been corrected.

Corrected:

**Author contributions**

Conceptualization: P.L., F.L.; Methodology: P.L.; Formal analysis: P.L.; Investigation: P.L., S.G.; Writing - original draft: P.L., F.L.; Writing - review & editing: F.L.; Supervision: F.L.; Project administration: F.L.; Funding acquisition: F.L.

Original:

**Author contributions**

Conceptualization: P.L., F.L.; Methodology: P.L.; Formal analysis: P.L.; Investigation: P.L.; Writing - original draft: P.L., F.L.; Writing - review & editing: F.L.; Supervision: F.L.; Project administration: F.L.; Funding acquisition: F.L.

Both the online and PDF versions of the paper have been corrected.

The authors apologise to readers for this error.

