# Supplementary material for: Heme-deficient primitive red blood cells induce HSPC ferroptosis by altering iron homeostasis during zebrafish embryogenesis
Source: Development. 2023 Jun 16;150(20):dev201690. doi: 10.1242/dev.201690 (PMC10281259; doi:10.1242/dev.201690)
Supplement: Supplementary information [file develop-150-201690-s1.pdf]

Figure S1

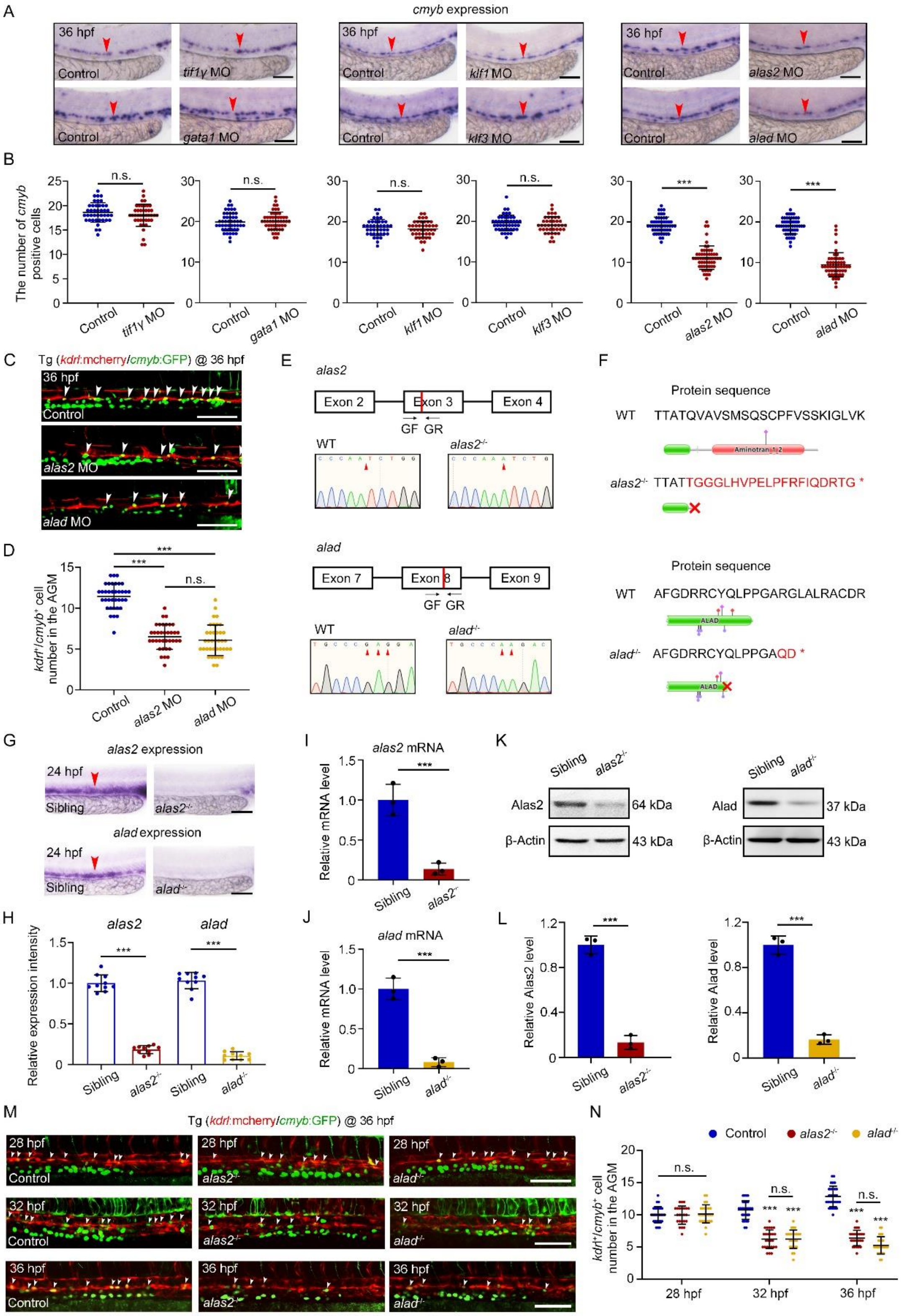

A
cmyb expression
36 hpf
Control
tif1γ MO
gata1 MO
klf1 MO
klf3 MO
alas2 MO
alad MO
B
The number of cmyb positive cells
n.s.
***
Control
C
Tg (kdrl:mcherry/cmyb:GFP) @ 36 hpf
D
kdrl+/cmyb+ cell number in the AGM
E
alas2
Exon 2
Exon 3
Exon 4
GF GR
WT
alas2-/-
alad
Exon 7
Exon 8
Exon 9
alad-/-
F
Protein sequence
WT TTATQVAVSMSQSCPFVSSKIGLVK
alas2-/- TTATTGGGLHVPELPFRFIQDRTG *
WT AFGDRRCYQLPPGARGLALRACDR
alad-/- AFGDRRCYQLPPGAQD *
G
alas2 expression
24 hpf
Sibling
alad expression
H
Relative expression intensity
I
alas2 mRNA
Relative mRNA level
J
alad mRNA
K
Alas2
64 kDa
β-Actin
43 kDa
Alad
37 kDa
L
Relative Alas2 level
Relative Alad level
M
28 hpf
32 hpf
36 hpf
N
28 hpf
32 hpf
36 hpf

Fig. S1. Screening and characterization of zebrafish embryos deficient in alas2 and alad. (**A**) Expression of HSPC marker cmyb in control, tif1γ-, gata1- ,klf1-, klf3-, alas2-, and alad-morphants at 36 hpf examined by WISH. The AGM region for marker gene-positive cell counting is denoted in red arrowheads. **(B)** Quantification of the *cmyb*-positive HSPCs in (A). Data are mean ± s.d; n=3 experimental replicates.. **(C)** Confocal imaging shows the *kdrl*$^{+}$/*cmyb*$^{+}$ HSPCs in the AGM region of control, *alas2*- and *alad*-morphants at 36 hpf. The *kdrl*$^{+}$/*cmyb*$^{+}$ HSPCs are denoted in white arrowheads. **(D)** Quantification of the *kdrl*$^{+}$/*cmyb*$^{+}$ HSPCs in (C). Data are mean ± s.d; n=3 experimental replicates. **(E-F)** Schematic representation for the generation of mutant alleles of *alas2* and *alad* using the CRISPR/Cas9 system. The mutated sites of *alas2* and *alad* are in the 3rd and 8th exon, respectively. The nucleotide sequences in (E) show that early termination of the translation occurred in both mutants. The red letters denote the mutated protein sequence and red asterisks indicate the stop codon in (F). **(G)** Expression of *alas2* and *alad* in *alas2*$^{-/-}$, *alad*$^{-/-}$ and their siblings at 24 hpf examined by WISH. The ICM regions are denoted in red arrowheads. **(H)** Quantification of the relative expression intensity of *alas2* or *alad* in (G), respectively. Data are mean ±s.d; n (embryos)=10. **(I-J)** Relative mRNA level of *alas2* (I) and *alad* (J) in *alas2*$^{-/-}$, *alad*$^{-/-}$ and their own siblings at 36 hpf examined by qRT-PCR, respectively. Data are mean ±s.d; n=3 experimental replicates. **(K)** Western-blotting detected the protein level of Alas2 and Alad in *alas2*$^{-/-}$, *alad*$^{-/-}$ and their own siblings at 36 hpf, respectively. **(L)** Quantification of protein levels in (K). Protein levels are analyzed by using 8-bit-gray analysis (Gel-Pro analyzer). Data are mean ± s.d; n=3 experimental replicates. **(M)** Confocal imaging shows the *kdrl*$^{+}$/*cmyb*$^{+}$ cells in control, *alas2*$^{-/-}$ and *alad*$^{-/-}$ at 28, 32 and 36 hpf, respectively. The *kdrl*$^{+}$/*cmyb*$^{+}$ HSPCs in AGM region are denoted in white arrowheads. **(N)** Quantification of the *kdrl*$^{+}$/*cmyb*$^{+}$ cells in (M). Data are mean ±s.d; n=3 experimental replicates. Number of samples are indicated, statistical analyses in B, H-J and L: Mann-Whitney non-parametric U test, in D and N: one-way ANOVA, Tukey's multiple comparisons, *P*-values: n.s.=not significant, ****P*<0.001. Scale bars in A, C, G and M: 100 µm.

Development • Supplementary information

Figure S2

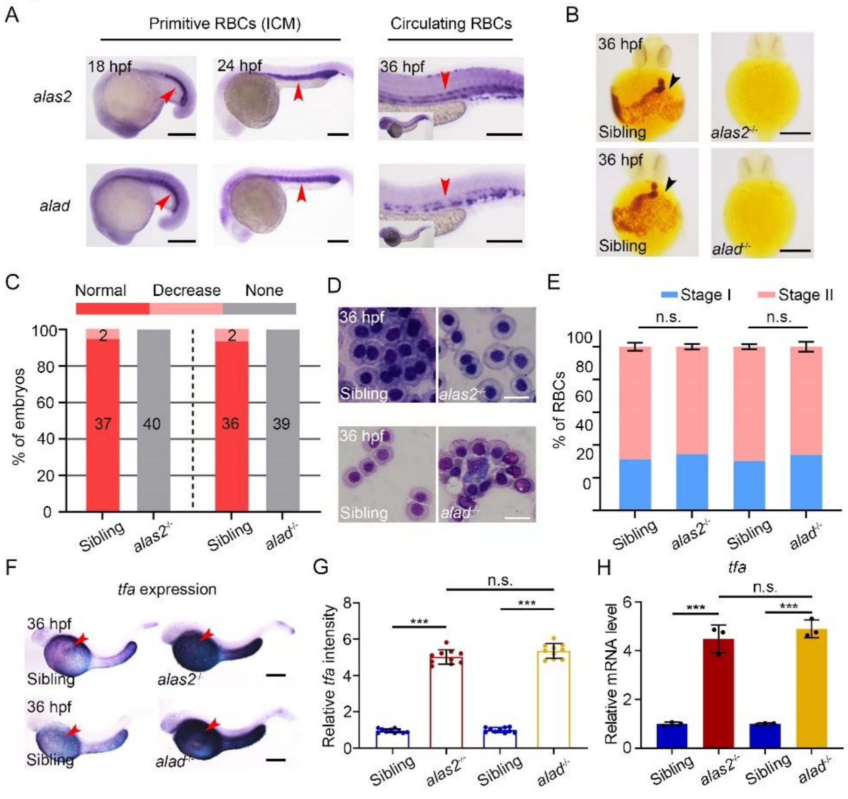

Fig. S2. Impaired iron metabolism in heme-deficient primitive RBC condition. (A) Expression patterns of *alas2* and *alad* WT embryos at 18, 24 and 36 hpf examined by WISH. The *alas2* and *alad* enriched regions are denoted in red arrowheads. **(B)** O-dianisidine staining in *alas2*$^{-/-}$, *alad*$^{-/-}$ and their siblings at 36 hpf. Black arrowheads denote the region of hemoglobin enriched primitive RBCs. **(C)** Cumulative results of hemoglobin content in (B). Number of embryos with different hemoglobin content are denoted in each column. **(D)** Representative Wright-Giemsa staining pictures of RBCs in *alas2*$^{-/-}$, *alad*$^{-/-}$ and their siblings at 36 hpf. **(E)** Statistical analysis of the percentage of RBCs at different maturation stages in (D). Stage I: basophilic erythroblast; Stage II: polychromatophilic erythroblast. Number of RBCs: n (sibling of *alas2*$^{-/-}$)=166, n (*alas2*$^{-/-}$)=170, n (sibling of *alad*$^{-/-}$)=173, n (*alad*$^{-/-}$)=176. Data are mean ± s.d; n=3 experimental replicates. **(F)** The expression of *tfa* is detected by WISH in *alas2*$^{-/-}$, *alad*$^{-}$/- and their siblings at 36 hpf. The yolk regions are denoted in red arrowheads. **(G)** Statistical analysis of the relative *tfa* expression in (G) by ImageJ. Data are mean ±s.d; n=10 (one-way ANOVA, Tukey's multiple comparisons). **(H)** Whole embryo of *alas2*$^{-/-}$, *alad*$^{-/-}$ and their siblings are collected for detecting the expression of *tfa* by qRT-PCR at 36 hpf. Data are mean ± s.d; n=3 experimental replicates. Number of samples are indicated, statistical analyses in G: one-way ANOVA, Tukey's multiple comparisons, in E: two-way ANOVA, Sidak's multiple comparisons, P-values: n.s.=not significant, ***P<0.001. Scale bars in A and F: 250 μm; in B: 200 μm; in D: 10 μm.

Figure S3

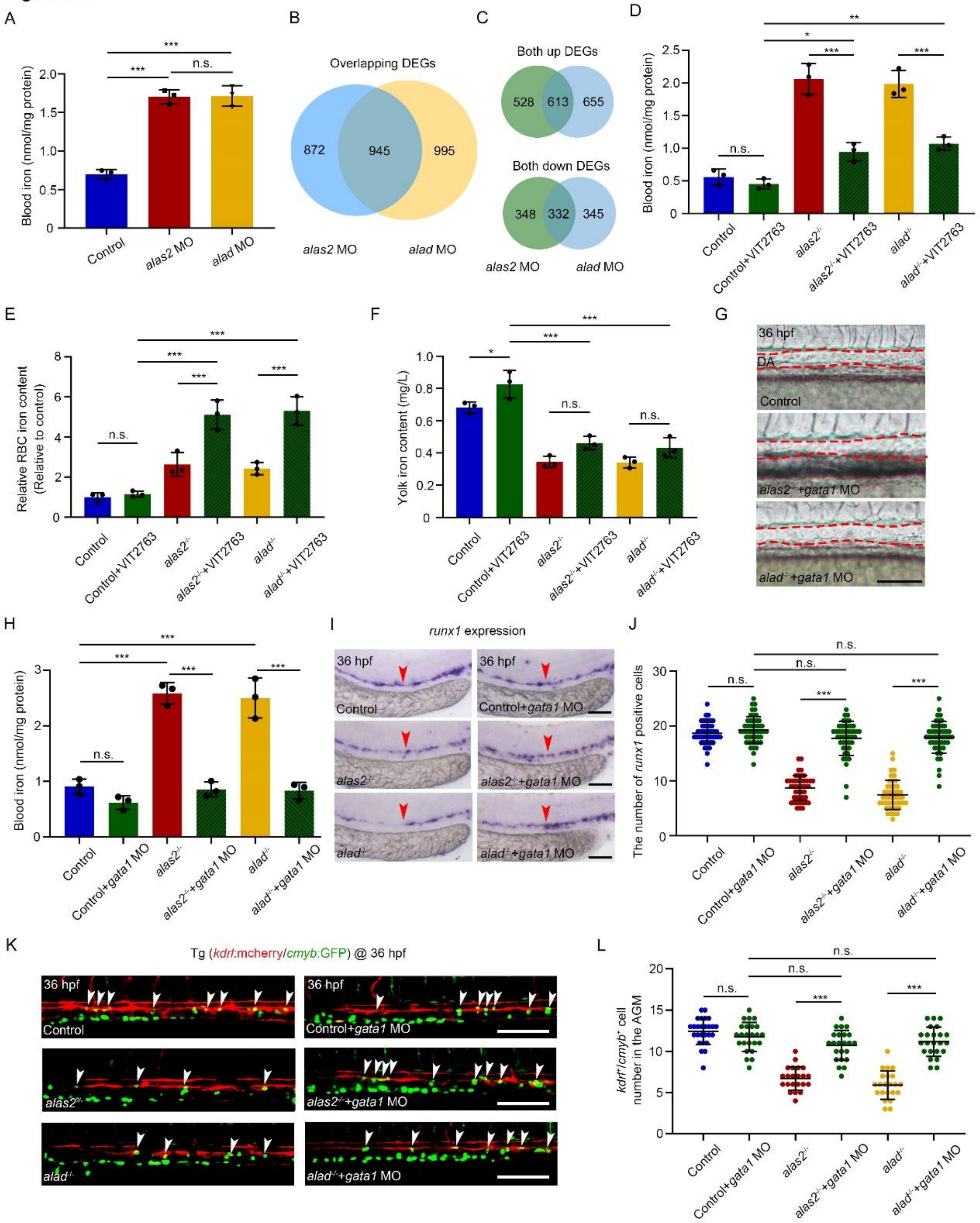

Fig. S3. Blood IOL and HSPC defects are abnormal RBC-dependent. (A) Quantification of the blood iron content by Iron colorimetric assay kit in control, *alas2*-and *alad*-morphant embryos at 36 hpf. Data are mean ± s.d; n=3 experimental replicates. **(B-C)** Venn diagram of the identified overlapping total (B) or up- and down-regulated (C) differential expressed genes (DEGs) in primitive RBCs of *alas2*-and *alad*-morphants at 36 hpf. **(D)** Quantification of the blood iron content in control, *alas2*$^{-/-}$ and *alad*$^{-/-}$ with or without VIT2763 treatment at 36 hpf.

Development • Supplementary information

Data are mean ±s.d; n=3 experimental replicates. **(E)** Quantification of the RBC iron content in control, *alas2*$^{-/-}$ and *alad*$^{-/-}$ with or without VIT2763 treatment at 36 hpf. Data are mean ± s.d; n=3 experimental replicates. **(F)** Quantification of the yolk iron content in control, *alas2*$^{-/-}$ and *alad*$^{-/-}$ with or without VIT2763 treatment at 36 hpf. Data are mean ± s.d; n=3 experimental replicates. **(G)** Brightfield images of blood flow in control, and *gata1* morpholino-injected *alas2*$^{-/-}$ and *alad*$^{-/-}$ at 36 hpf. Blood flow in DA regions is denoted in red dashed lines. **(H)** Quantification of the blood iron level in control, *alas2*$^{-/-}$ and *alad*$^{-/-}$ with or without *gata1* morpholino injection at 36 hpf. Data are mean ± s.d; n=3 experimental replicates. **(I)** Expression of HSPC marker gene *runx1* in control, *alas2*$^{-/-}$ and *alad*$^{-/-}$ with or without *gata1* morpholino injection at 36 hpf examined by WISH. The AGM region for marker gene positive cell counting is denoted in red arrowheads. **(J)** Quantification of the *runx1* positive HSPCs in (I). Data are mean ± s.d; n=3 experimental replicates. **(K)** Confocal imaging shows the *kdrl*$^{+}$/*cmyb*$^{+}$ HSPCs in control, *alas2*$^{-/-}$ and *alad*$^{-/-}$ with or without *gata1* morpholino injection at 36 hpf, respectively. The *kdrl*$^{+}$/*cmyb*$^{+}$ cells in the AGM region are denoted in white arrowheads. **(L)** Quantification of the *kdrl*$^{+}$/*cmyb*$^{+}$ cells in (K). Data are mean ± s.d; n=3 experimental replicates. Number of samples are indicated, statistical analyses in A, D, E, F, H, J and L: one-way ANOVA, Tukey's multiple comparisons, *P*-values: n.s.=not significant, **P*<0.05, ***P*<0.01, ****P*<0.001. Scale bars in G, I and K: 100 μm.

Development • Supplementary information

Figure S4

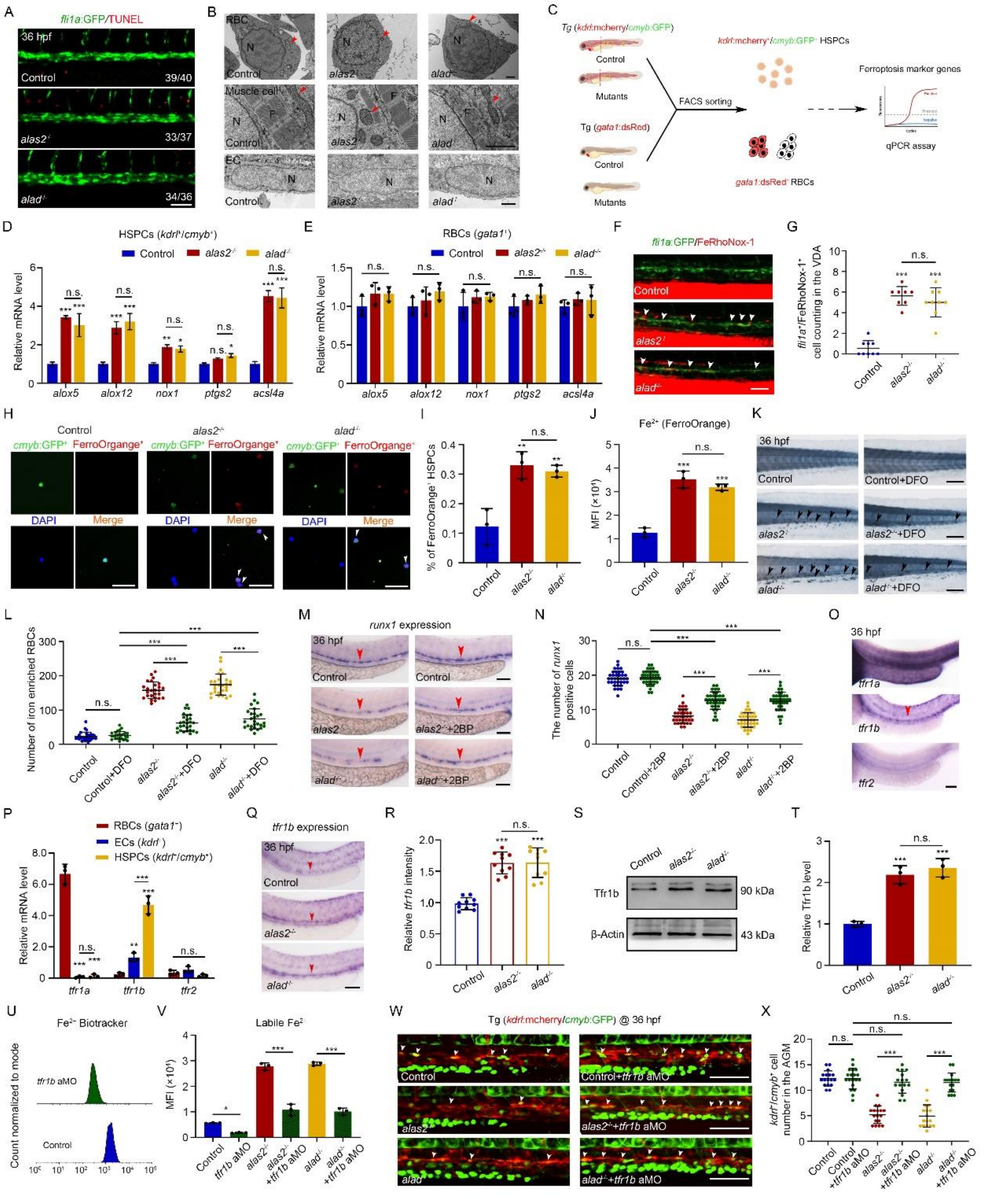

Fig. S4. Excessive iron promotes HSPC ferroptosis in a primitive RBC-dependent manner. (A) Confocal imaging shows no co-localization of *fli1a*:GFP$^+$ cells with TUNEL signaling in *alas2*$^{-/-}$, *alad*$^{-/-}$ and their siblings at 36 hpf. **(B)** The TEM view of RBCs (top panel), muscle cells (middle panel) and ECs (bottom panel) in control, *alas2*$^{-/-}$ and *alad*$^{-/-}$ at 36 hpf. Mitochondria in each panel denotes in red arrowheads. (Abbreviations: N, nucleus, F, muscle fiber). **(C-E)** Relative mRNA level of ferroptotic activators in the flow cytometric sorted *kdrl*$^+$/*cmyb*$^+$ HSPCs (D) or *gata1*$^+$ RBCs (E) of control, *alas2*$^{-/-}$ and *alad*$^{-/-}$ at 36 hpf examined by qRT-PCR, respectively. Data are mean ±s.d; n=3 experimental replicates. **(F)** Confocal imaging shows the co-localization of fli1a:GFP+

and FeRhoNox-1^{+} cells in the VDA of control, *alas2*$^{-/-}$ and *alad*$^{-/-}$ at 36 hpf. The *fli1a*$^{+}$/FeRhoNox-1^{+} cells are denoted in white arrowheads. **(G)** Quantification of the *fli1a*$^{+}$/FeRhoNox-1^{+} cells in (F). Data are mean ± s.d; n=3 experimental replicates. **(H)** Representative fluorescence image shows the co-localization of *cmyb*:GFP^{+} and FerroOrange^{+} cells in *alas2*$^{-/-}$ and *alad*$^{-/-}$ at 36 hpf. The *cmyb*$^{+}$/ FerroOrange^{+} cells are denoted in white arrowheads. **(I)** Statistical analysis of the percentage of the FerroOrange^{+} HSPCs in control, *alas2*$^{-/-}$ and *alad*$^{-/-}$ at 36 hpf measured by flow-cytometry. Data are mean ±s.d; n=3 experimental replicates. **(J)** Quantification of MFI of Fe^{2+} level in HSPCs of control, *alas2*$^{-/-}$ and *alad*$^{-/-}$ measured by FerroOrange staining. Data are mean ± s.d; n=3 experimental replicates. **(K)** Representative brightfield images of DAB-enhanced iron staining in control, *alas2*$^{-/-}$ and *alad*$^{-/-}$ with or without DFO treatment at 36 hpf. The region for iron enriched RBC counting is denoted in black arrowheads. **(L)** Quantification of the DAB-enhanced iron staining positive cells in (K). Data are mean ±s.d; n=3 experimental replicates. **(M)** Expression of HSPC marker *runx1* in control, *alas2*$^{-/-}$ and *alad*$^{-/-}$ with or without 2BP treatment (10 μM) at 36 hpf examined by WISH. The AGM regions for marker gene positive cell counting are denoted in red arrowheads. **(N)** Quantification of the *runx1*-positive HSPCs in (M). Data are mean ± s.d; n=3 experimental replicates. **(O)** Expression patterns of *tfr1a*, *tfr1b* and *tfr2* in wild-type embryos at 36 hpf examined by WISH. The AGM region are denoted in red arrowheads. **(P)** Relative mRNA level of *tfr1a*, *tfr1b* and *tfr2* in the flow cytometric sorted RBCs (*gata1*$^{+}$), ECs (*kdrl*$^{+}$) and HSPCs (*kdrl*$^{+}$/*cmyb*$^{+}$) of wild-type embryos at 36 hpf examined by qRT-PCR, respectively. Data are mean ± s.d; n=3 experimental replicates. **(Q)** Expression of *tfr1b* in the AGM regions of control, *alas2*$^{-/-}$ and *alad*$^{-/-}$ at 36 hpf examined by WISH. The AGM regions are denoted in red arrowheads. **(R)** Statistical analysis of the relative *tfr1b* expression in (Q) by ImageJ. Data are mean ±s.d; n (embryos)=10. **(S)** Western-blotting detect the protein level of Tfr1b in flow-cytometric sorted HSPCs of control, *alas2*$^{-/-}$, *alad*$^{-/-}$ at 36 hpf, respectively. (30,000 HSPCs are sorted in each group). **(T)** Quantification of protein levels in (S). Protein levels are analyzed by using 8-bit-gray analysis (Gel-Pro analyzer). Data are mean ±s.d; n=3 experimental replicates. **(U)** Representative flow-cytometric histogram of the Fe^{2+} level in sorted HSPCs of control and *tfr1b* aMO injected embryos at 36 hpf measured by Fe^{2+} biotracker dye. **(V)** Quantification of MFI of labile Fe^{2+} level in (U). Data are mean ± s.d; n=3 experimental replicates. **(W)** Confocal imaging shows the kdrl+/cmyb+ HSPCs in control, alas2-/- and alad-/- with or without tfr1b aMO injection at 36 hpf. (X) Quantification of the HSPCs in (W). Data are mean ± s.d; n=3 experimental replicates. Number of samples are indicated, statistical analyses in D, E, G, I, J, L, N, P, R, T, V and X: one-way ANOVA, Tukey's multiple comparisons, P-values: n.s.=not significant, *P<0.05, **P<0.01, ***P<0.001. Scale bars in A, K, M, O, Q and W: 100 μm; in B: 1 μm; in F and H: 50 μm.

Development • Supplementary information

Figure S5

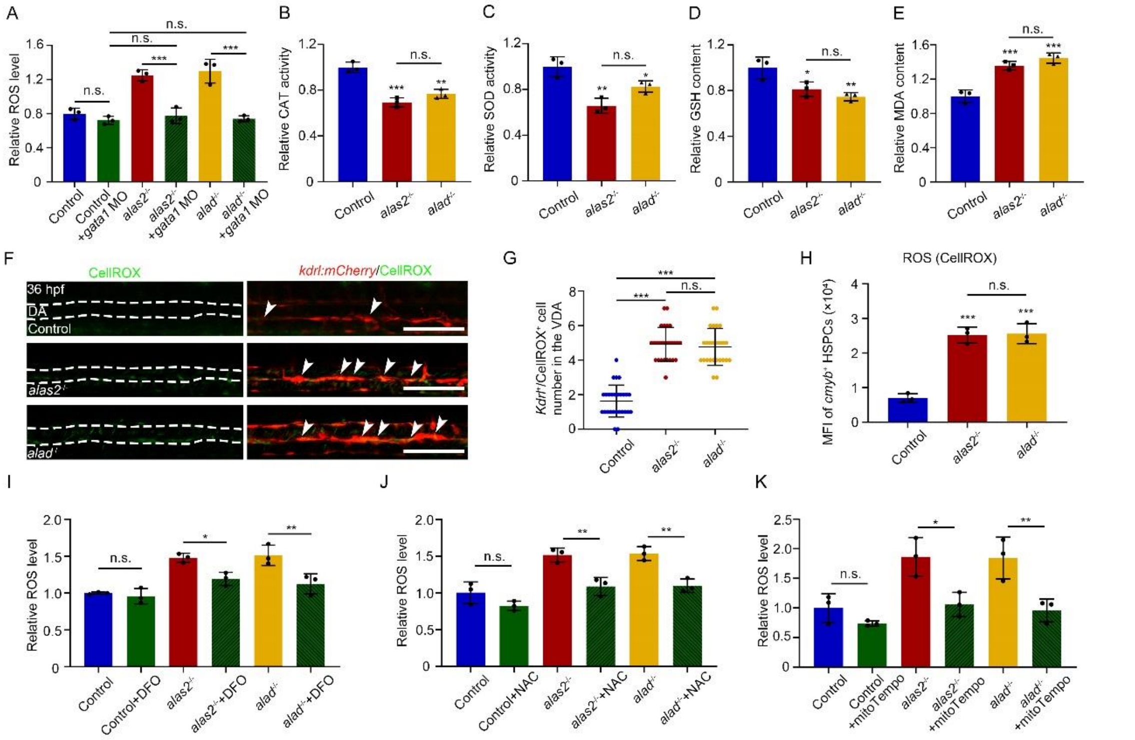

Fig. S5. Oxidative stress is increased in *alas2* and *alad* mutants. (A) Relative ROS level in control, *alas2*$^{-/-}$ and *alad*$^{-/-}$ with or without *gata1* morpholino injection at 36 hpf measured by DCFH-DA staining. Data are mean ±s.d; n=3 experimental replicates. **(B-E)** The relative activity of CAT (B), SOD (C), and the content of GSH (D), MDA (E) are measured by commercial kits in control, *alas2*$^{-/-}$ and *alad*$^{-/-}$ at 36 hpf. Data are mean ± s.d; n=3 experimental replicates. **(F)** Confocal imaging shows the co-localization of *kdrl*:mCherry^{+} and CellROX^{+} cells in the VDA of control, *alas2*$^{-/-}$ and *alad*$^{-/-}$ at 36 hpf. The DA regions are denoted in white dashed lines, and the *kdrl*$^{+}$/CellROX^{+} cells are denoted in white arrowheads. **(G)** Quantification of the *kdrl*$^{+}$/CellROX^{+} cells in (F). Data are mean ±s.d; n=3 experimental replicates. **(H)** Quantification of MFI of ROS level in HSPCs of control, *alas2*$^{-/-}$ and *alad*$^{-/-}$ measured by CellROX staining. Data are mean ±s.d; n=3 experimental replicates. **(I)** Relative ROS level in control, *alas2*$^{-/-}$ and *alad*$^{-/-}$ with or without DFO treatment at 36 hpf measured by DCFH-DA staining. Data are mean ±s.d; n=3 experimental replicates. **(J)** Relative ROS level in control, *alas2*$^{-/-}$ and *alad*$^{-/-}$ with or without NAC treatment at 36 hpf measured by DCFH-DA staining. Data are mean ±s.d; n=3 experimental replicates. **(K)** Relative ROS level in control, *alas2*$^{-/-}$ and *alad*$^{-/-}$ with or without mitoTempo treatment at 36 hpf measured by DCFH-DA staining. Data are mean ±s.d; n=3 experimental replicates. Number of samples are indicated, statistical analyses in A-E and G-K: one-way ANOVA, Tukey's multiple comparisons, P-values: n.s.=not significant, *P<0.05, **P<0.01, ***P<0.001. Scale bars in F: 100 μm.

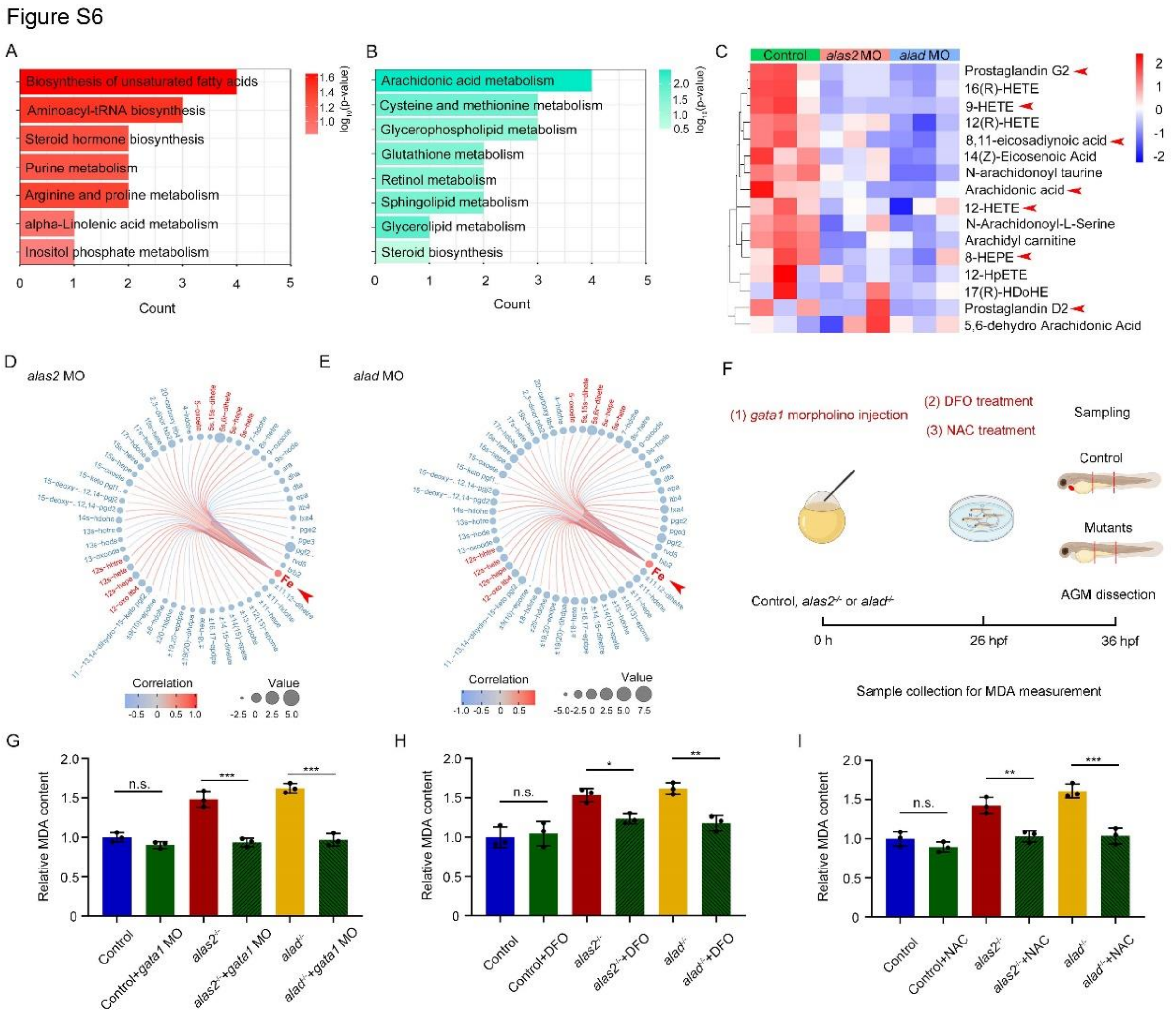

Fig. S6. Oxidative lipids produce through both enzymatic and fenton reaction pathways in *alas2* and *alad* deficient embryos. (A-B) The metabolic pathway analyses showing the enrichment of up- (A) and down-regulated (B) overlapping pathways in *alas2*- and *alad*-morphants. **(C)** Heatmap analysis of DRMs in overlapping metabolic pathways. Metabolites involved in “Arachidonic acid metabolism” are denoted in red arrowheads. **(D-E)** Correlation analyses of iron with differentially regulated oxidative lipids in *alas2*- (D) and *alad*-morphants (E). A positive correlation is labeled in red strings and negative in blue. The intensity of correlation is represented as the dot size. Iron (Fe) is highlighted in red. **(F)** Schematic workflow for the MDA content measurement after *gata1* morpholino injection, DFO treatment and NAC treatment in the AGM regions of control, *alas2*- and *alad*-morphant at 36 hpf. **(G-I)** Relative MDA content in control, *alas2*$^{-/-}$ and *alad*$^{-/-}$ with or without *gata1* morpholino injection (G), DFO treatment (H), and NAC treatment (I). Data are mean ±s.d; n=3 experimental replicates. Number of samples are indicated, statistical analyses in G-I: one-way ANOVA, Tukey’s multiple comparisons, *P*-values: n.s.=not significant, **P*<0.05, ***P*<0.01, ****P*<0.001.

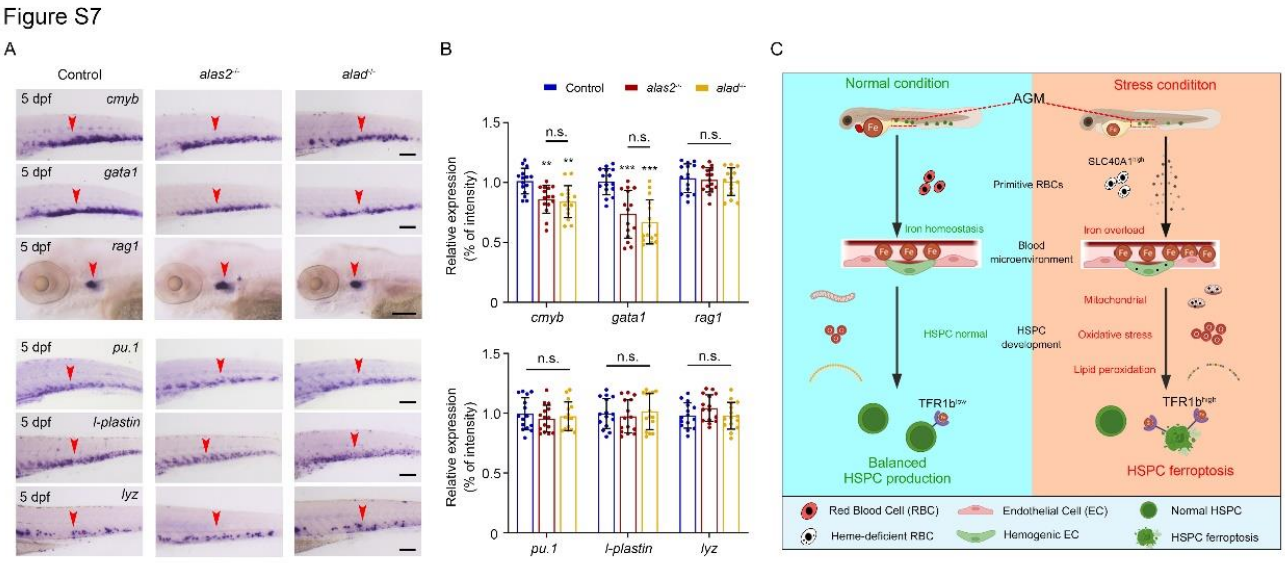

Fig. S7. The generation of definitive RBCs was impaired in *alas2* or *alad* mutants. (A) Expression of HSPC marker *cmyb*, RBC marker *gata1*, lymphocyte marker *rag1* and myelocyte markers *pu.1*, *l-plastin* and *lyz* in control, *alas2*$^{-/-}$ and *alad*$^{-/-}$ at 5 dpf examined by WISH. The CHT or thymus regions for marker gene expression level quantification are denoted in red arrowheads. **(B)** Statistical analysis of the relative *slc40a1* expression in (A) by ImageJ. Data are mean ± s.d; n (embryos)=15. **(C)** Schematic diagram of HSPC ferroptosis under heme-deficient primitive RBC induced iron overload stress conditions. Left panel shows that HSPC development is normal under blood homeostasis, and results in balanced HSPC generating. Right panel shows that in *alas2* and *alad* mutants, primitive RBCs lead to blood iron overload via Slc40a1. The development of tfr1bhigh erythrocyte-biased HSPC is impaired by iron-induced oxidative stress, subsequently lead to HSPC ferroptosis. Number of samples are indicated, statistical analyses in B: one-way ANOVA, Tukey's multiple comparisons, *P*-values: n.s.=not significant, ***P*<0.01, ****P*<0.001. Scale bars, 100 µm.

Table S1. RNA-sequencing data of *gata1*$^{+}$ RBCs in control, *alas2*- and *alad*-morphants for generating RNA-seq related figures (Figures 3E-3G, S3B and S3C).

Click here to download Table S1

Table S2. Metabolomics data of control, *alas2*- and *alad*-morphants for generating related figures (Figures S6A-S6C).

Click here to download Table S2

Table S3. Oxidative lipidomics data of control, *alas2*- and *alad*-morphants for generating related figures (Figures 6B, 6C, S6D and S6C).

Click here to download Table S3

Table S4. All the oligo sequence used in this study.

Click here to download Table S4

Table S5. Values that were used to create all the graphs in the paper.

Click here to download Table S5

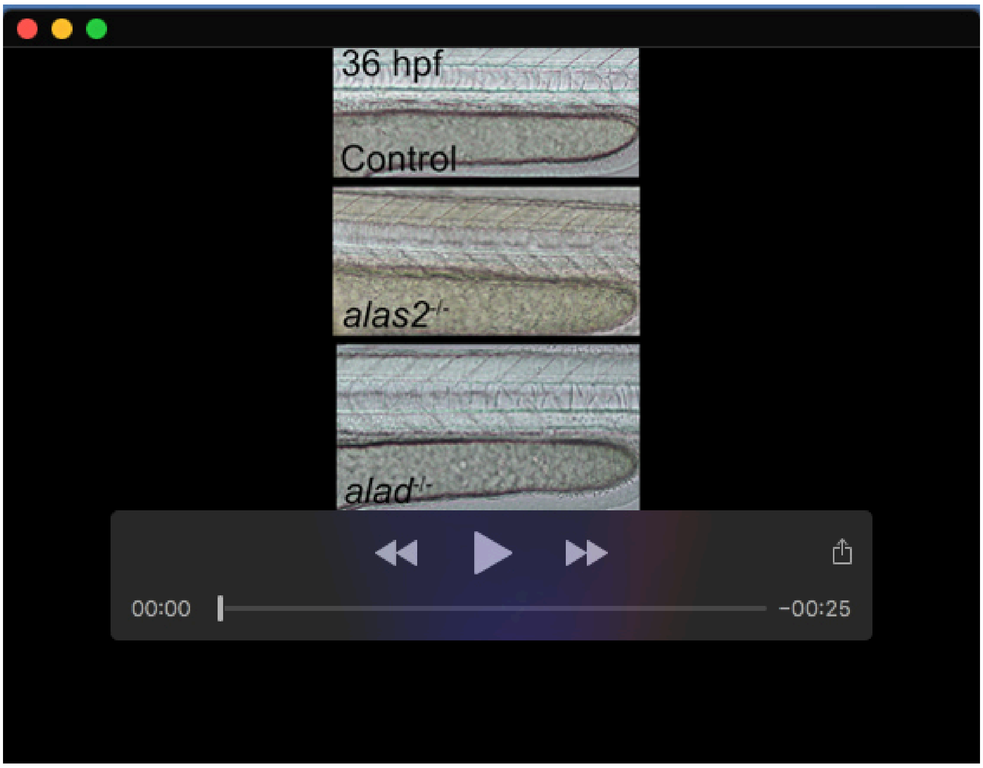

Movie 1. Blood flow in control, *alas2*$^{-/-}$ and *alad*$^{-/-}$.

Development • Supplementary information
