# Supplementary material for: Heme-deficient primitive red blood cells induce HSPC ferroptosis by altering iron homeostasis during zebrafish embryogenesis
Source: Development. 2023 Jun 16;150(20):dev201690. doi: 10.1242/dev.201690 (PMC10281259; doi:10.1242/dev.201690)
Supplement: Supplementary file 1 [file develop-150-201690_review_history.pdf]

Heme-deficient primitive red blood cells induce HSPC ferroptosis by altering iron homeostasis during zebrafish embryogenesis

Peng Lv and Feng Liu
DOI: 10.1242/dev.201690

Editor: Hanna Mikkola

Review timeline

Original submission:	7 February 2023
Editorial decision:	22 March 2023
First revision received:	2 May 2023
Accepted:	11 May 2023

Original submission

First decision letter

MS ID#: DEVELOP/2023/201690

MS TITLE: Heme-deficient primitive red blood cells induce HSPC ferroptosis by altering iron homeostasis during zebrafish embryogenesis

AUTHORS: Peng Lv and Feng Liu

I have now received all the referees' reports on the above manuscript, and have reached a decision. The referees' comments are appended below, or you can access them online: please go to BenchPressand click on the 'Manuscripts with Decisions' queue in the Author Area.

As you will see, the referees express considerable interest in your work, but have some significant criticisms and recommend a substantial revision of your manuscript before we can consider publication. If you are able to revise the manuscript along the lines suggested, which may involve further experiments, I will be happy receive a revised version of the manuscript. Your revised paper will be re-reviewed by one or more of the original referees, and acceptance of your manuscript will depend on your addressing satisfactorily the reviewers' major concerns. Please also note that Development will normally permit only one round of major revision. If it would be helpful, you are welcome to contact us to discuss your revision in greater detail. Please send us a point-by-point response indicating your plans for addressing the referee’s comments, and we will look over this and provide further guidance.

Please attend to all of the reviewers' comments and ensure that you clearly highlight all changes made in the revised manuscript. Please avoid using 'Tracked changes' in Word files as these are lost in PDF conversion. I should be grateful if you would also provide a point-by-point response detailing how you have dealt with the points raised by the reviewers in the 'Response to Reviewers' box. If you do not agree with any of their criticisms or suggestions please explain clearly why this is so.

© 2023. Published by The Company of Biologists under the terms of the Creative Commons Attribution License (https://creativecommons.org/licenses/by/4.0/).

Reviewer 1

Advance summary and potential significance to field

In this manuscript, Lv and Liu present interesting data that demonstrates how impairments in heme biosynthesis in primitive RBCs can result in ferroptosis in HSPCs in a cell non-autonomous manner. They showed that heme synthesis deficient primitive RBCs can export excessive intracellular iron into the extracellular environment through iron transporter ferroportin/Slc40a1, which in turn is uptaken by HSPCs causing iron accumulation and ferroptosis in these cells in zebrafish. These findings could explain the HSPC defects that have been observed accompanying some alterations of primitive hematopoiesis, which helps tie together some previously unexplained phenomenon. Moreover, this work extends recent reports on ferroptosis sensitivity in the hematopoietic stem cell compartment (doi: 10.1016/j.cell.2023.01.020; 10.1101/2021.08.03.454947). This work presents another line of evidence that iron metabolism can be crucial for HSC/HSPC maintenance and provides insights into how neighboring cells can alter iron homeostasis.

Comments for the author

While the work presented here is interesting and important to present to the community, some issues should be resolved with further experiments and analyses. In particular, some additional mechanistic studies and assessment of protein levels, where possible, would significantly help in interpreting some of the findings.

I have the following specific comments:

- Figure S1C: the author should specify which color represents which protein marker (akin to what is shown in Figure 1J).

- In Figure 1C it is unclear what the authors are trying to statistically compare. It appears that they are trying to convey the concept that virtually all controls have normal blood and all alas2 and alad KO have colorless blood, but the way this data is presented is unusual (e.g. having two bars with significance asterisks made it difficult to understand what the authors are trying to say here by just looking at the data).
- In Figure S1M, the authors should specify which color represents which protein marker (akin to what is shown in Figure 1J).

- The finding that alas2 or alad KO RBCs are phenotypically normal is quite interesting and counterintuitive. Have the authors examined the hemoglobin content of the primitive RBCs? Are the animals with alas2 or alad KO viable until adulthood? Could the authors speculate why this might be the case?

- In Fig S2G it is questionable that the authors use tfa expression as an iron storage marker. The cited reference did not imply that tfa expression can be used as an intracellular iron storage marker. Instead, they used apo-transferrin in an iron efflux experiment because of its iron binding activity. There is no firm evidence showing that tfa is necessary for iron storage, unless the authors do additional positive control experiments where they treat the animal embryo in a way that they know there will be an increase in iron storage. Indeed, tfa primarily binds labile irons, which will not impact iron stores. To examine stores, fth or ftl might be better, or a simple iron staining could be done here. Also, in Fig S2H, it is again confusing what the authors are trying to quantify. It is very arbitrary to quantify staining signals as just an “increase” or “normal”. An quantitative signal detection method would be more objective here.

- In Fig 3I it is again confusing what the authors are trying to quantify. It is very arbitrary to quantify staining signals as just “increase” or “normal”, as discussed in the prior comment.

- In Fig 4A and lines 211-212 the authors have misinterpreted what the referenced literature reported. In Dixon et al. paper that first described ferroptosis, they clearly indicated that the ferroptotic cells do not harbor chromatin condensation, which is indicative of apoptosis. They specifically state:

© 2023. Published by The Company of Biologists under the terms of the Creative Commons Attribution License (https://creativecommons.org/licenses/by/4.0/).

“tumor cells treated with erastin exhibited none of the characteristic morphologic features associated with staurosporine (STS)-induced apoptosis (e.g., chromatin condensation and margination), hydrogen peroxide (H2O2)-induced necrosis (e.g., cytoplasmic and organelle swelling, plasma membrane rupture), or rapamycin-induced autophagy (e.g., formation of double-membrane enclosed vesicles). The lone distinctive morphological feature of erastin-treated cells involved mitochondria that appeared smaller than normal with increased membrane density, consistent with our previous report (Yagoda et al., 2007) ”

In the subsequent review papers referenced in line 211-212, the authors of those review papers have also only referenced the Dixon paper and clearly stated that ferroptotic cells do not harbor chromatin condensation phenotype, or any other phenotypes observed in other forms of cell death. Therefore, it will be important to re-evaluate these images carefully and quantification of mitochondrial alterations, as has been done in other recent reports, will be important (doi: 10.1016/j.cell.2023.01.020).

- In Fig 4D it is counterintuitive why the cells upregulate the expression of drivers of ferroptosis and downregulate expression of suppressors in an environment that is already iron-overloaded/toxic. In other cases when ferroptosis is induced, ferroptosis protective genes will be significantly upregulated. Could these changes be assessed at the protein level? Some regulation might occur at the level of translation and this could explain some of the observed counterintuitive changes in gene expression.

- Iron exists both in the form of redox inactive ferric (Fe^{3+}) and redox active ferrous (Fe^{2+}) forms and so the data in Figure 4D needs to be carefully assessed. The dyes used here selectively bind and detect labile ferrous iron only and cannot assess ferric iron concentration, whereas the iron detection methods used previously detect overall iron levels, including both ferric and ferrous irons. The authors should make clear claims about what they are trying to measure. Ferric iron is usually quite stable in the cell and does not normally change. Is this the case in the cells assessed here? Moreover, the dyes used here are very sensitive and detect even traces of ferrous iron, so it is risky to describe the cells as simple as positive vs. negative. It is recommended that the authors report mean fluorescence intensity here and include a negative and positive controls.

- In Figure 4K the authors again did not include a description of which color represents which protein. There are series of similar images throughout the manuscript and sometimes they have a description on top of the images and sometimes they do not. The authors should ensure label consistency across the entire manuscript for easier interpretation of the data.

- Based on what is shown in Figure S4M-O, can the authors propose a mechanism why HSPCs do not suppress tfr1b expression in response to an iron-toxic environment? Did the authors try to look at transferrin receptor protein levels?

- The function of mitochondria ROS in ferroptosis induction is very controversial. In early reports on ferroptosis, it appears that mitochondria ROS was not relevant (doi: 10.1016/j.cell.2012.03.042). Later it was shown that mitochondria ROS does increase sometimes with ferroptosis, particularly with SLC7A11 inhibition (doi: 10.1016/j.molcel.2018.10.042). From the mitoSOX staining, can the authors explain where these mitochondria ROS come from and whether this is relevant to the ferroptosis phenotype? Can the authors rescue the phenotype by using mitochondria specific antioxidants such as mitoTempo, which will increase the confidence that mitochondria ROS is important in inducing ferroptosis under this context.

- Given that GSH is the intracellular antioxidant fuel used by GPX4, can the authors postulate a reason why it is reduced in Figure S5D? Did the cells show lower Slc7a11 protein levels, and therefore, lower cystine to glutamate exchange?

- Given that NAC is a very general antioxidant, a more specific ferroptosis inhibitor beyond an iron chelator should be employed to rescue the observed phenotype.

© 2023. Published by The Company of Biologists under the terms of the Creative Commons Attribution License (https://creativecommons.org/licenses/by/4.0/).

Reviewer 2

Advance summary and potential significance to field

The manuscript by Lv et al utilized the alas2- and alad-deficient embryos to study the functional role of primitive RBCs in definitive HSPCs production. They found that heme-deficient RBCs caused iron-overload in the blood which in turn could induce the ferroptosis of nascent HSPCs. Mechanistically, upregulation of the iron exporter slc40a1 in RBCs and the transferrin receptors tf1rb in HSPCs could account for the iron over-release and uptake, respectively. The iron overload induces the excessive ROS, which causes the lipid peroxidation to trigger HSPC ferroptosis. Overall, the work is well-organized and the findings that primitive RBCs-iron-ROS-lipid peroxidation axis regulates HPSCs ferroptosis will broaden the understanding of definitive HSPC generation.

Comments for the author

There are a couple of concerns which may further improve the quality of the manuscript if the authors could address.

1. The authors suggested that the enrichment of transferrin receptor tf1rb expression contributed to the ferroptosis of the HSPCs. To support this conclusion the authors need to demonstrate that depletion or knockdown of tf1rb rescues the HSPC phenotype.

2. In Fig.7, the authors performed primary and secondary transplantation assays to argue that under iron overload stress conditions, erythrocyte-biased HSPCs undergo ferroptosis, while the other lineages-biased HSPCs (Fig.7D, E) together with the self-renewal and multi-lineage differentiation of HSCs appear normal (Fig.7G,H). Although the authors claimed that “the other lineages appeared not changed” (Line 351-353), there seems significant difference in myeloid and lymphoid lineages as well (Fig. 7D, E), suggesting that the other lineage-biased HSPCs are also affected. Additional evidence (perhaps WISH analysis to examine the expression of different lineage markers at 5-6 dpf embryos) will be required to confirm their conclusion.

First revision

Author response to reviewers' comments

We are grateful to the editors and reviewers for careful reading our manuscript and providing many insightful comments to our manuscript. We have performed a number of experiments and analyses to improve the quality of our manuscript. The more detailed responses to reviewers’ comments are shown below.

Reviewer 1 Advance Summary and Potential Significance to Field:

In this manuscript, Lv and Liu present interesting data that demonstrates how impairments in heme biosynthesis in primitive RBCs can result in ferroptosis in HSPCs in a cell non-autonomous manner. They showed that heme synthesis deficient primitive RBCs can export excessive intracellular iron into the extracellular environment through iron transporter ferroportin/mi40a1, which in turn is uptaken by HSPCs causing iron accumulation and ferroptosis in these cells in zebrafish. These findings could explain the HSPC defects that have been observed accompanying some alterations of primitive hematopoiesis, which helps tie together some previously unexplained phenomenon. Moreover, this work extends recent reports on ferroptosis sensitivity in the hematopoietic stem cell compartment (doi:10.1016/j.cell.2023.01.020; 10.1101/2021.08.03.454947). This work presents another line of evidence that iron metabolism can be crucial for HSC/HSPC maintenance and provides insights into how neighboring cells can alter iron homeostasis.

© 2023. Published by The Company of Biologists under the terms of the Creative Commons Attribution License (https://creativecommons.org/licenses/by/4.0/).

Reviewer 1 Comments for the Author:

While the work presented here is interesting and important to present to the community, some issues should be resolved with further experiments and analyses. In particular, some additional mechanistic studies and assessment of protein levels, where possible, would significantly help in interpreting some of the findings.

We thank the reviewer for the constructive and detailed comments. Recent study showed that human HSC is sensitive to ferroptosis due to the absence of histone deubiquitinase, MYSM1 (Zhao et al., 2023). In this study, we also found that developing HSPCs are sensitive to ferroptosis under primitive RBC- induced iron-overload conditions. To strengthen our findings, we detected the protein level of several ferroptosis-related markers (such as Fth1, Gpx4, Slc7a11, and Tfr1b) in flow-cytometric sorted *kdrl*:mcherry^{+}/*cmyb*:GFP^{+} HSPCs of *alas2* or *alad* mutants at 36 hpf. Additionally, we performed anti- ferroptosis treatment to rescue HSPC defects with mitoTempo or Ferrostatin-1 (Fer-1). Moreover, we reanalyzed the ferrous iron level in the HSPCs, and revaluated the ferroptosis features of HSPCs.

I have the following specific comments:

Figure S1C: the author should specify which color represents which protein marker (akin to what is shown in Figure 1J).

Response 1: Thanks for the suggestion. We have added the detailed information to show the color representations in the revised Figures throughout the manuscript (Please see the revised **Fig. 4S, Fig. S1C, M** and **Fig. S4W**).

In Figure 1C it is unclear what the authors are trying to statistically compare. It appears that they are trying to convey the concept that virtually all controls have normal blood and all alas2 and alad KO have colorless blood, but the way this data is presented is unusual (e.g. having two bars with significance asterisks made it difficult to understand what the authors are trying to say here by just looking at the data).

Response 2: Thanks for this comment. To avoid misunderstanding, we have revised the figures by referring to a previous study (Rossmann et al., 2021). We have removed the statistical comparisons and only displayed the percentage of phenotype distribution (revised **Fig. 1C**), and similarly the revision has been made in revised **Fig. S2C** (please see in revised **Fig. S2C**).

Note: We have removed unpublished data provided for the referees in confidence

In Figure S1M, the authors should specify which color represents which protein marker (akin to what is shown in Figure 1J).

Response 3: We have added the detailed information to show the color representations in the revised

Figure S1M, please also see in **Response 1**.

The finding that alas2 or alad KO RBCs are phenotypically normal is quite interesting and counterintuitive. Have the authors examined the hemoglobin content of the primitive RBCs? Are the animals with alas2 or alad KO viable until adulthood? Could the authors speculate why this might be the case?

Response 4: Thanks for this thoughtful comment. In our data, we have showed that the hemoglobin was undetectable in *alas2* or *alad* mutants at 36 hpf by O-Dianisidine staining (**Fig. S2B**). To further evaluate the hemoglobin content during primitive erythropoiesis, we have performed time-course study (at 25, 28 and 32 hpf) in the *alas2* or *alad* mutants and their siblings by O-Dianisidine staining. The result showed that RBC hemoglobin biosynthesis pathway was

© 2023. Published by The Company of Biologists under the terms of the Creative Commons Attribution License (https://creativecommons.org/licenses/by/4.0/).

absolutely blocked in the *alas2* or *alad* knockout embryos at around 28 hpf (The initiation stage of hemoglobin biosynthesis) (**Response Fig. 1**).

Note: We have removed unpublished data provided for the referees in confidence

Response Fig. 1. Time-course study of RBC hemoglobin biosynthesis. Representative images of hemoglobin content in the circulating RBCs of *alas2* or *alad* mutants and their siblings detected by O- dianisidine staining. The number of embryos was indicated. Scale bars: 200 µm.

Zebrafish *alas2* or *alad* homozygous mutants are lethal and die within 3 weeks post-fertilization

(**Response Fig. 2**).

Note: We have removed unpublished data provided for the referees in confidence

Response Fig. 2. Survival rate of *alas2* or *alad* mutants. The survival rate of control, *alas2* and *alad* mutants at 21 days post-fertilization (dpf). The number of embryos in each group was indicated.

We speculated that the oxygen deficit is a leading cause of mortality in the *alas2* or *alad* mutants, due to the RBC heme-deficiency. However, zebrafish embryos can absorb enough oxygen by means of passive diffusion via the skin in the earlier developmental stages (Chavez et al., 2016; Gore et al., 2012).

In Fig S2G it is questionable that the authors use tfa expression as an iron storage marker. The cited reference did not imply that tfa expression can be used as an intracellular iron storage marker. Instead, they used apo-transferrin in an iron efflux experiment because of its iron binding activity. There is no firm evidence showing that tfa is necessary for iron storage, unless the authors do additional positive control experiments where they treat the animal embryo in a way that they know there will be an increase in iron storage. Indeed, tfa primarily binds labile irons, which will not impact iron stores. To examine stores, fth or ftl might be better, or a simple iron staining could be done here.

Response 5: Thanks for this critical comment. We agree that it is not appropriate to utilize *tfa* mRNA level to indicate yolk total iron storge, therefore, we have revised the relevant descriptions in our revised manuscript (please see in **Line 139-143**).

Then, we have showed that the yolk iron content was significantly decreased in the *alas2* or *alad* mutants by ICP-MS assay (**Fig. 2C**). To confirm this, we further examined yolk iron content by iron colorimetric assay kit (revised **Fig. 2D**). Both results demonstrate that yolk iron content is remarkably decreased in the *alas2* or *alad* mutants.

Note: We have removed unpublished data provided for the referees in confidence

Next, we have checked the expression pattern of *fth1a* (probe ID: fq18c10) or *fthl27* (probe ID: cb141) in The Zebrafish Information Network database (ZFIN, https://zfin.org/); however, neither of them showed the yolk-enriched expression pattern.

Also, in Fig S2H, it is again confusing what the authors are trying to quantify. It is very arbitrary to quantify staining signals as just an “increase” or “normal”. An quantitative signal detection method would be more objective here.

Response 6: We have quantified the relative intensity of *tfa* expression by Image-J (revised **Fig. S2G**). Consistently, we have made the similar revision on the Figure S1H (please see in revised **Fig. S1H**) and Figure 3I (please see in revised **Fig. 3I**).

Note: We have removed unpublished data provided for the referees in confidence

In Fig 3I it is again confusing what the authors are trying to quantify. It is very arbitrary to

© 2023. Published by The Company of Biologists under the terms of the Creative Commons Attribution License (https://creativecommons.org/licenses/by/4.0/).

quantify staining signals as just “increase” or “normal”, as discussed in the prior comment.

Response 7: We have quantified the relative intensity of *slc40a1* expression by ImageJ (revised **Fig. S3I**). Please also see **Response 6**.

Note: We have removed unpublished data provided for the referees in confidence

In Fig 4A and lines 211-212 the authors have misinterpreted what the referenced literature reported. In Dixon et al. paper that first described ferroptosis, they clearly indicated that the ferroptotic cells do not harbor chromatin condensation, which is indicative of apoptosis. They specifically state: “tumor cells treated with erastin exhibited none of the characteristic morphologic features associated with staurosporine (STS)-induced apoptosis (e.g., chromatin condensation and margination), hydrogen peroxide (H2O2)-induced necrosis (e.g., cytoplasmic and organelle swelling, plasma membrane rupture), or rapamycin-induced autophagy (e.g., formation of double-membrane enclosed vesicles). The lone distinctive morphological feature of erastin-treated cells involved mitochondria that appeared smaller than normal with increased membrane density, consistent with our previous report (Yagoda et al., 2007) ”

In the subsequent review papers referenced in line 211-212, the authors of those review papers have also only referenced the Dixon paper and clearly stated that ferroptotic cells do not harbor chromatin condensation phenotype, or any other phenotypes observed in other forms of cell death. Therefore, it will be important to re-evaluate these images carefully and quantification of mitochondrial alterations, as has been done in other recent reports, will be important (doi: 10.1016/j.cell.2023.01.020).

Response 8: Thanks for pointing out this critical aspect of this study. Firstly, we have reread these cited papers and other studies related to morphological changes of ferroptotic cells. Most of the studies demonstrated that chromatin condensation was not a typic feature in the ferroptotic cells, while a recent review claimed that ferroptotic cells might display a necrosis-like morphological changes, including moderate chromatin condensation (Tang et al., 2021).

To avoid misleading, we carefully reanalyzed the TEM data of ferroptotic HSPCs, especially the morphological change in chromatin, and we found that the occurring of moderate chromatin condensation was not-significant in the HSPCs of *alas2* or *alad* mutants (**Response Fig. 3A, B**). Based on this, we removed the chromatin condensation related figures (please see in revised **Fig. 4B**) and descriptions (please see in **Line 205 and 208, Line 210-213**).

Note: We have removed unpublished data provided for the referees in confidence

Response Fig. 3. Chromatin condensation was not a key feature in ferroptotic HSPCs of *alas2* or *alad* mutants. (A) The TEM view of HSPC with normal chromatin in control, *alas2* or *alad* mutants. (N: nucleus). (B) Statistical analysis of the percentage of normal chromatin in ferroptotic HSPC of *alas2* or *alad* mutants. Data are mean ±s.d; (one-way ANOVA, Tukey’s multiple comparisons). Number of HSPCs were indicated, *P*-values: n.s.=not significant. Scale bars in A: 1 μm.

Then, referring to the recent work (Zhao et al., 2023), we have quantified the phenotypes of mitochondrial alterations and membrane disruption in the HSPCs of control, *alas2* or *alad* mutants, respectively (revised **Fig. 4C-F**).

Note: We have removed unpublished data provided for the referees in confidence

In Fig 4D it is counterintuitive why the cells upregulate the expression of drivers of ferroptosis and downregulate expression of suppressors in an environment that is already iron-overloaded/toxic. In other cases when ferroptosis is induced, ferroptosis protective genes will be significantly upregulated. Could these changes be assessed at the protein level? Some regulation might occur at the level of translation and this could explain some of the observed counterintuitive changes in gene expression.

© 2023. Published by The Company of Biologists under the terms of the Creative Commons Attribution License (https://creativecommons.org/licenses/by/4.0/).

Response 9: Thanks for this critical comment. It has been reported that some ferroptosis driver-encoding genes were up-regulated in ferroptotic cells, such as *acsl4*, *ptgs2* (Stockwell, 2022), and *nox1* (Zhu et al., 2021). Consistently, we observed the up-regulation of these genes in the *alas2* or *alad* mutants, and we speculated that the up-regulation of *nox1*, *alox5* and *alox12* might be one of the leading causes of ROS and peroxidative lipid over-production (please see in **Fig. S5A and Fig. 6C**).

To further study the regulation of ferroptosis-protective system, we used several antibodies to detect the protein level of Fth1, Gpx4, and Slc7a11 in the flow-cytometric sorted HSPCs (30,000 *kdrl*:mcherry$^+$/*cmyb*:GFP$^+$ cells) (revised **Fig. 4G**).

Note: We have removed unpublished data provided for the referees in confidence

We screened out three antibodies that can recognize these targeted proteins in zebrafish, and the results showed that all the proteins were significantly decreased in the HSPCs of *alas2* or *alad* mutants (revised **Fig. 4H, I**).

Note: We have removed unpublished data provided for the referees in confidence

Iron exists both in the form of redox inactive ferric (Fe3+) and redox active ferrous (Fe2+) forms and so the data in Figure 4D needs to be carefully assessed. The dyes used here selectively bind and detect labile ferrous iron only and cannot assess ferric iron concentration, whereas the iron detection methods used previously detect overall iron levels, including both ferric and ferrous irons. The authors should make clear claims about what they are trying to measure. Ferric iron is usually quite stable in the cell and does not normally change. Is this the case in the cells assessed here? Moreover, the dyes used here are very sensitive and detect even traces of ferrous iron, so it is risky to describe the cells as simple as positive vs. negative. It is recommended that the authors report mean fluorescence intensity here and include a negative and positive controls.

Response 10: Thanks for this suggestion. Previous studies demonstrated that the ferrous iron was the main trigger of ferroptosis (Jenkins et al., 2020; Xing et al., 2022), and the ferrous iron level was also carefully analyzed in a recent study (Zhao et al., 2023). Therefore, we here measured the redox-active ferrous iron level in the HSPCs of *alas2* or *alad* mutants by FeRhoNox-1 and FerroOrange staining, respectively. To clarify the form of iron detected in the HSPCs, we have added the relevant descriptions in the revised manuscript (please see in **Line 226-237**).

Next, as suggested, we have reanalyzed the ferrous iron level in each group by using mean fluorescence intensity (MFI) (revised **Fig. 4P and S4J**).

Note: We have removed unpublished data provided for the referees in confidence

Additionally, we have set both negative (100 µM DFO treatment) and positive (1 mM $FeSO_4$ treatment) controls and measured the labile ferrous iron in the HSPCs of *alas2* or *alad* mutants with another fluorescent dye, Fe^{2+} Biotracker dye (sigma, SCT037). As expected, we found that the ferrous iron level was remarkably elevated in the HSPCs of *alas2* or *alad* mutants (revised **Fig. 4K, L**).

Note: We have removed unpublished data provided for the referees in confidence

In Figure 4K the authors again did not include a description of which color represents which protein. There are series of similar images throughout the manuscript and sometimes they have a description on top of the images and sometimes they do not. The authors should ensure label consistency across the entire manuscript for easier interpretation of the data.

Response 11: We have added the detailed information to show the color representations in the revised

Figure 4S, please also see **Response 1**.

© 2023. Published by The Company of Biologists under the terms of the Creative Commons Attribution License (https://creativecommons.org/licenses/by/4.0/).

Based on what is shown in Figure S4M-O, can the authors propose a mechanism why HSPCs do not suppress tfr1b expression in response to an iron-toxic environment? Did the authors try to look at transferrin receptor protein levels?

Response 12: Under normal physiological condition, the *tfr* mRNA level is negatively correlated with the intracellular iron level. However, we found that the *tfr1b* was significantly up-regulated in the HSPCs of *alas2* or *alad* mutants, implying that the transcriptional regulation of iron transporter was altered. There are two possible explanations: (1) Dysregulation in iron regulation proteins (IRPs) (Martelli et al., 2015); (2) Erythropoietin (EPO) induced *tfr* up-regulation under some RBC-related disease conditions (Coulon et al., 2011; Paulson, 2011).

To confirm the increasement of Tfr1b at protein level, we screened out one TFR1 (also called CD71 in mammals) antibodies that can recognize zebrafish Tfr1b in the flow-cytometric sorted HSPCs (30,000 *kdrl*:mcherry^{+}/*cmyb*:GFP^{+} cells). The results showed that the protein level of Tfr1b was also remarkably elevated in the HSPCs of *alas2* or *alad* mutants (revised **Fig. S4S, T**).

Note: We have removed unpublished data provided for the referees in confidence

The function of mitochondria ROS in ferroptosis induction is very controversial. In early reports on ferroptosis, it appears that mitochondria ROS was not relevant (doi: 10.1016/j.cell.2012.03.042). Later it was shown that mitochondria ROS does increase sometimes with ferroptosis, particularly with SLC7A11 inhibition (doi: 10.1016/j.molcel.2018.10.042). From the mitoSOX staining, can the authors explain where these mitochondria ROS come from and whether this is relevant to the ferroptosis phenotype? Can the authors rescue the phenotype by using mitochondria specific antioxidants such as mitoTempo, which will increase the confidence that mitochondria ROS is important in inducing ferroptosis under this context.

Response 13: Thanks for this thoughtful suggestion. Based on our data, we think the most possible explanation for mitochondria ROS elevation might be the accumulation of ferrous iron. Previous studies have demonstrated that iron can drive cellular or mitochondrial ROS production (Gao et al., 2019; Huang et al., 2017). In our data, we also found that the elevated ROS could be evidently restored by DFO treatment **(Fig. S5I**).

Then, we further confirmed the ROS elevation in the HSPCs of *alas2* or *alad* mutants by analyzing the MFI of ROS intensity in the *cmyb*$^{+}$ HSPCs (AGM region) by MitoSOX and CellROX (revised **Fig. 5D** and **Fig. S5H**).

Note: We have removed unpublished data provided for the referees in confidence

To study whether mitochondrial ROS is tightly correlated to HSPC ferroptosis, we treated the embryos (control, *alas2*, or *alad* mutants) with mitoTempo. The results showed that mitochondrial ROS level was evidently reduced in the *alas2* or *alad* mutants with mitoTempo treatment (revised **Fig. S5K**), and that the HSPC defect was also rescued efficiently (revised **Fig. 5G-J**).

Note: We have removed unpublished data provided for the referees in confidence

Given that GSH is the intracellular antioxidant fuel used by GPX4, can the authors postulate a reason why it is reduced in Figure S5D? Did the cells show lower Slc7a11 protein levels, and therefore, lower cystine to glutamate exchange?

Response 14: Thanks for this thoughtful comment. We speculated that there might be two reasons for the reduction of GSH level. (1) The cells with decreased Slc7a11 protein; (2) The cells with reduced activity of γ-glutamylcysteine synthetase (γ-GCS), a rate-limiting enzyme in GSH biosynthesis pathway. To validate these hypotheses, we have performed western-blot and measured the γ-GCS activity in the flow-cytometric sorted HSPCs. We found that the Scl7a11 (also known as xCT) was significantly decreased in the HSPCs of *alas2* or *alad* mutans (please also see in **Response 9**, and revised **Fig. 4H, I**). However, we found that the activity of γ-GCS was unchanged in the HSPCs among control, *alas2* or *alad* mutants (**Response Fig. 4**). Taken together, we suggest that the

© 2023. Published by The Company of Biologists under the terms of the Creative Commons Attribution License (https://creativecommons.org/licenses/by/4.0/).

reduction of GSH in the HSPCs of *alas2* or *alad* mutants results from the decreased GSH substrate transporter, Slc7a11.

Note: We have removed unpublished data provided for the referees in confidence

Response Fig. 4. The relative activity of γ-GCS was unchanged in HSPCs of *alas2* or *alad* mutants. The relative activity of γ-GCS was measured by commercial kits in control, *alas2*$^{-/-}$ and *alad*$^{-/-}$ at 36 hpf. Data are mean ±s.d; n=3 experimental replicates (one-way ANOVA, Tukey's multiple comparisons).

Given that NAC is a very general antioxidant, a more specific ferroptosis inhibitor beyond an iron chelator should be employed to rescue the observed phenotype.

Response 15: Thanks for this suggestion. We have employed another well-studied ferroptosis inhibitor, Ferrostatin-1 (Fer-1), to rescue the HSPC defects in the *alas2* or *alad* mutants. The results showed that the HSPC defects can be efficiently rescued by Fer-1 treatment (revised **Fig. 4U-X**).

Note: We have removed unpublished data provided for the referees in confidence

Reviewer 2 Advance Summary and Potential Significance to Field:

The manuscript by Lv et al utilized the alas2- and alad-deficient embryos to study the functional role of primitive RBCs in definitive HSPCs production. They found that heme-deficient RBCs caused iron- overload in the blood which in turn could induce the ferroptosis of nascent HSPCs. Mechanistically, upregulation of the iron exporter slc40a1 in RBCs and the transferrin receptors tf1rb in HSPCs could account for the iron over-release and uptake, respectively. The iron overload induces the excessive ROS, which causes the lipid peroxidation to trigger HSPC ferroptosis. Overall, the work is well-organized and the findings that primitive RBCs-iron-ROS-lipid peroxidation axis regulates HPSCs ferroptosis will broaden the understanding of definitive HSPC generation.

Reviewer 2 Comments for the Author:

There are a couple of concerns which may further improve the quality of the manuscript if the authors could address.

1. The authors suggested that the enrichment of transferrin receptor tf1rb expression contributed to the ferroptosis of the HSPCs. To support this conclusion, the authors need to demonstrate that depletion or knockdown of tf1rb rescues the HSPC phenotype.

Response 16: Thanks for the supportive and constructive comments. To further analyze the role of *tfr1b* in HSPC ferroptosis, we have designed the *tfr1b* ATG-morpholino (aMO) to block the translation of *tfr1b* by referring to a previous report (Fraenkel et al., 2009). We firstly confirmed the efficacy of *tfr1b* aMO mediated knockdown in the HSPCs by detecting the ferrous iron level (Fe^{2+} Biotracker dye) (revised **Fig. S4U, V**).

Note: We have removed unpublished data provided for the referees in confidence

Then, we found that the HSPC defect can be evidently rescued in the *alas2* or *alad* mutant with *tfr1b* aMO injection (revised **Fig. S4W, X**).

Note: We have removed unpublished data provided for the referees in confidence

2. In Fig.7, the authors performed primary and secondary transplantation assays to argue that under iron overload stress conditions, erythrocyte-biased HSPCs undergo ferroptosis, while the other lineages- biased HSPCs (Fig.7D, E) together with the self-renewal and multi-lineage differentiation of HSCs appear normal (Fig.7G,H). Although the authors claimed that "the other lineages appeared not changed" (Line 351-353), there seems significant difference in myeloid and lymphoid lineages as well (Fig. 7D, E), suggesting that the other lineage-biased HSPCs are also affected. Additional

© 2023. Published by The Company of Biologists under the terms of the Creative Commons Attribution License (https://creativecommons.org/licenses/by/4.0/).

evidence (perhaps WISH analysis to examine the expression of different lineage markers at 5-6 dpf embryos) will be required to confirm their conclusion.

Response 17: Thanks for this critical comment. We apologize for this inaccurate description and have corrected it in the revised manuscript (please see in **Line 358-360**). We transplanted an equal number of HSPCs, while the compositions of donor HSPCs were varied, such as control (containing balanced subtypes of HSPCs) and mutants (we suppose a decreased in erythrocyte-biased HSPCs).

To further confirm this, we have detected the multilineage differentiation of HSPCs with different lineage markers (HSPCs: *cmyb*; erythroid cells: *gata1*; myeloid cells: *pu.1*, *l-plastin* and *lyz*; lymphoid cells: *rag1*) in the control, *alas2* and *alad* mutants at 5 dpf by WISH. The results showed that the number of HSPCs and erythroid cells were evidently decreased, while other lineages were unchanged (revised **Fig. S7A, B**).

Note: We have removed unpublished data provided for the referees in confidence

Reference

Chavez, M. N., Aedo, G., Fierro, F. A., Allende, M. L. and Egana, J. T. (2016). Zebrafish as an Emerging Model Organism to Study Angiogenesis in Development and Regeneration. *Front Physiol* **7**, 56.

Coulon, S., Dussiot, M., Grapton, D., Maciel, T. T., Wang, P. H., Callens, C., Tiwari, M. K., Agarwal, S., Fricot, A., Vandekerckhove, J., et al. (2011). Polymeric IgA1 controls erythroblast proliferation and accelerates erythropoiesis recovery in anemia. *Nat Med* **17**, 1456-1465.

Fraenkel, P. G., Gibert, Y., Holzheimer, J. L., Lattanzi, V. J., Burnett, S. F., Dooley, K. A., Wingert, R. A. and Zon, L. I. (2009). Transferrin-a modulates hepcidin expression in zebrafish embryos. *Blood* **113**, 2843-2850.

Gao, M., Yi, J., Zhu, J., Minikes, A. M., Monian, P., Thompson, C. B. and Jiang, X. (2019). Role of Mitochondria in Ferroptosis. *Mol Cell* **73**, 354-363 e353.

Gore, A. V., Monzo, K., Cha, Y. R., Pan, W. and Weinstein, B. M. (2012). Vascular development in the zebrafish. *Cold Spring Harb Perspect Med* **2**, a006684.

Huang, H., Chen, J., Lu, H., Zhou, M., Chai, Z. and Hu, Y. (2017). Iron-induced generation of mitochondrial ROS depends on AMPK activity. *Biometals* **30**, 623-628.

Jenkins, N. L., James, S. A., Salim, A., Sumardy, F., Speed, T. P., Conrad, M., Richardson, D. R., Bush, A. I. and McColl, G. (2020). Changes in ferrous iron and glutathione promote ferroptosis and frailty in aging Caenorhabditis elegans. *Elife* **9**.

Martelli, A., Schmucker, S., Reutenauer, L., Mathieu, J. R. R., Peyssonnaux, C., Karim, Z., Puy, H., Galy, B., Hentze, M. W. and Puccio, H. (2015). Iron regulatory protein 1 sustains mitochondrial iron loading and function in frataxin deficiency. *Cell Metab* **21**, 311-323. **Paulson, R. F.** (2011). Erythropoiesis lagging? pIgA1 steps in to assist Epo. *Nat Med* **17**, 1346-1348.

Rossmann, M. P., Hoi, K., Chan, V., Abraham, B. J., Yang, S., Mullahoo, J., Papanastasiou, M., Wang, Y., Elia, I., Perlin, J. R., et al. (2021). Cell-specific transcriptional control of mitochondrial metabolism by TIF1gamma drives erythropoiesis. *Science* **372**, 716-721. **Stockwell, B. R.** (2022). Ferroptosis turns 10: Emerging mechanisms, physiological functions, and therapeutic applications. *Cell* **185**, 2401-2421.

Tang, D., Chen, X., Kang, R. and Kroemer, G. (2021). Ferroptosis: molecular mechanisms and health implications. *Cell Res* **31**, 107-125.

Xing, G., Meng, L., Cao, S., Liu, S., Wu, J., Li, Q., Huang, W. and Zhang, L. (2022). PPARalpha alleviates iron overload-induced ferroptosis in mouse liver. *EMBO Rep* **23**, e52280.

Zhao, J., Jia, Y., Mahmut, D., Deik, A. A., Jeanfavre, S., Clish, C. B. and Sankaran, V. G. (2023). Human hematopoietic stem cell vulnerability to ferroptosis. *Cell* **186**, 732-747 e716.

Zhu, L., Yang, F., Wang, L., Dong, L., Huang, Z., Wang, G., Chen, G. and Li, Q. (2021). Identification the ferroptosis-related gene signature in patients with esophageal adenocarcinoma. *Cancer Cell Int* **21**, 124.

© 2023. Published by The Company of Biologists under the terms of the Creative Commons Attribution License (https://creativecommons.org/licenses/by/4.0/).

Second decision letter

MS ID#: DEVELOP/2023/201690

MS TITLE: Heme-deficient primitive red blood cells induce HSPC ferroptosis by altering iron homeostasis during zebrafish embryogenesis

AUTHORS: Peng Lv and Feng Liu
ARTICLE TYPE: Research Article

I am happy to tell you that your manuscript has been accepted for publication in Development, pending our standard ethics checks.

Reviewer 1

Advance summary and potential significance to field

Deciphers the regulation of ferroptosis of HSPCs due to altered iron handling

Comments for the author

The authors have adequately addressed all of my concerns. I did notice some minor grammatical errors, so a final proofread could be valuable.

Reviewer 2

Advance summary and potential significance to field

The authors have addressed all the concerns I have, and it is now suitable for publication.

Comments for the author

I have no further questions.

© 2023. Published by The Company of Biologists under the terms of the Creative Commons Attribution License (https://creativecommons.org/licenses/by/4.0/).
